# Antiviral Strategies Targeting Enteroviruses: Current Advances and Future Directions

**DOI:** 10.3390/v17091178

**Published:** 2025-08-28

**Authors:** Michelle Felicia Lee, Seng Kong Tham, Chit Laa Poh

**Affiliations:** 1 Department of Biomedical Sciences, Faculty of Medical and Life Sciences, Sunway University, No.5, Jalan Universiti, Bandar Sunway 46150, Malaysia; michlee2311@gmail.com; 2 ALPS Global Holding Berhad, The ICON, East Wing Tower, No. 1, Jalan 1/68F, Off Jalan Tun Razak, Kuala Lumpur 50400, Malaysia; drtham@alpsmedical.com; 3 Nilai University, No. 1, Persiaran Universiti, Putra Nilai, Bandar Baru Nilai, Nilai 17800, Malaysia

**Keywords:** enteroviruses, antivirals, host-targeted antivirals, RNA interference, CRISPR-Cas systems, drug discovery and development

## Abstract

Enteroviruses, a diverse genus within the *Picornaviridae* family, are responsible for a wide range of human infections, including hand, foot, and mouth disease, respiratory disease, aseptic meningitis, encephalitis, myocarditis, and acute flaccid paralysis. Despite their substantial global health burden and the frequent emergence of outbreaks, no specific antiviral therapies are currently approved for clinical use against non-polio enteroviruses. This review provides a comprehensive overview of the current landscape of antiviral strategies targeting enteroviruses, including direct-acting antivirals such as capsid binders, protease inhibitors, and viral RNA polymerase inhibitors. We also examine the potential of host-targeting agents that interfere with virus–host interactions essential for replication. Emerging strategies such as immunotherapeutic approaches, RNA interference, CRISPR-based antivirals, and peptide-based antivirals are also explored. Furthermore, we address key challenges, including viral diversity, drug resistance, and limitations in preclinical models. By highlighting recent advances and ongoing efforts in antiviral development, this review aims to guide future research and accelerate the discovery of effective therapies against enterovirus infections.

## 1. Introduction

The *Enterovirus* (EV) genus, a member of the *Picornaviridae* family, consists of 13 species, of which 7 are known to infect humans. These include four EV species—EV-A, EV-B, EV-C, and EV-D—as well as three rhinovirus (RV) species—RV-A, RV-B, and RV-C. EVs that can cause human infections include EV-A71, Coxsackievirus A6 (CV-A6), CV-A10, and CV-A16 (EV-A); CV-B3, CV-B5, echoviruses, and CV-A9 (EV-B); poliovirus and CV-A21 (EV-C); and EV-D68 and EV-D70 (EV-D). Collectively, these viruses are responsible for a wide range of clinical manifestations, from mild illnesses to severe complications such as hand, foot, and mouth disease (HFMD), aseptic meningitis, encephalitis, myocarditis, acute flaccid paralysis, and respiratory diseases [1].

The EV genome is a positive-sense single-stranded RNA with a single open reading frame (ORF), flanked by 5′ and 3′ untranslated regions (UTRs) [2]. The ORF is translated into a single polyprotein that is proteolytically cleaved into three regions: P1, P2, and P3. P1 gives rise to the four structural capsid proteins VP1–VP4, which mediate viral entry by interacting with host cell receptors and uncoating the viral genome. P2 and P3 encode non-structural (NS) proteins (2A–2C and 3A–3D), which play essential roles in genome replication, polyprotein processing, and modulation of host cellular pathways [3]. Due to their involvement in critical stages of the viral life cycle, these viral proteins represent attractive targets for antiviral intervention.

Although most EV infections are self-limiting, certain serotypes can cause severe disease, particularly in infants, the elderly, and immunocompromised individuals. Over the past decade, EV-A71 and CV-A16 have caused major HFMD outbreaks across Asia, while EV-D68 has emerged as a respiratory pathogen associated with severe respiratory illness and acute flaccid myelitis, notably during the 2014 outbreak in North America [4,5,6]. These recurring and unpredictable outbreaks underscore the need for effective antiviral therapeutics. However, despite the considerable global health burden posed by non-polio enteroviruses, there are currently no approved antiviral therapies for clinical use.

In this review, we provide a comprehensive overview of the current strategies under investigation for the development of anti-EV therapeutics. We discuss direct-acting antivirals (DAAs), particularly capsid binders, protease inhibitors, and polymerase inhibitors, alongside host-targeting antivirals that disrupt virus–host interactions essential for replication. Additionally, we highlight immunotherapeutic approaches such as neutralizing monoclonal antibodies, intravenous immunoglobulin (IVIG), and type I interferons (IFNs). The emergence of novel technologies including RNA interference, CRISPR-based antivirals, and broad-spectrum antivirals will also be explored. Finally, we address challenges such as viral diversity, resistance, limitations of current preclinical models, and offer perspectives on the development of future therapeutic strategies.

## 2. Biology and Pathogenesis of Enteroviruses

### 2.1. Structure and Genome Organization of Enteroviruses

EVs are small, non-enveloped viruses with a positive-sense single-stranded RNA genome [7]. The virion, approximately 30 nm in diameter, possesses a naked icosahedral capsid exhibiting fivefold rotational symmetry and is formed by 60 identical protomers [8]. Each protomer comprises four structural proteins: VP1, VP2, VP3, and VP4. The outer surface of the capsid comprises VP1, VP2, and VP3, while VP4 is located internally, lining the capsid’s inner face in close proximity to the viral RNA. The capsid proteins VP1 to VP3 share a conserved architecture consisting of eight-stranded antiparallel β-barrels flanked by α-helices. Structural variability among different EVs arises primarily from differences in the loops that connect these secondary structures, contributing to serotype-specific antigenic features [1,3].

The surface topology of the EV capsid is characterized by distinctive morphological features. Star-shaped protrusions are formed by five copies of VP1 around the fivefold axes, while a canyon encircles these axes, formed by the junction of the “north rim” (VP1) and the “south rim” (VP2 and VP3). Additional surface structures include a prominent protrusion or “puff” generated by a loop in VP2 and a “knob” formed by a VP3 loop. The capsid also contains large depressions at the twofold axes. A conserved hydrophobic pocket lies beneath the canyon floor in VP1, typically occupied by a lipid-like molecule known as the “pocket factor”, which stabilizes the virion and plays a role in uncoating during cell entry [1,8,9]. While the amino acid residues lining this pocket are highly conserved, the identity of the pocket factor can vary between different EVs and may include sphingosine or other fatty acids, as observed in EV-A71 [10,11,12].

The EV genome spans approximately 7.4 kilobases and carries UTRs at both the 5′ and 3′ ends. The 5′ UTR contains a highly structured internal ribosome entry site (IRES), enabling cap-independent translation, and is covalently linked to a small viral protein, VPg, which is critical for initiating RNA replication. The 3′ UTR ends in a polyadenylated tail that is essential for genome stability and viral infectivity [1]. The viral genome encodes a single large open reading frame, which is translated into a polyprotein of approximately 2193 amino acids. This polyprotein undergoes proteolytic cleavage by viral proteases into three precursor regions: P1, P2, and P3 [13]. The P1 region is further processed into four structural proteins that form the viral capsid. The P2 and P3 regions yield seven NS proteins—2A, 2B, 2C, 3A, 3B (VPg), 3C (protease), and 3D (RNA-dependent RNA polymerase) [14].

These NS proteins are essential for viral replication and pathogenesis. They participate in the assembly of membranous replication organelles, facilitate the hijacking of host cell resources, and suppress host immune responses. Some of these proteins, such as 2A and 3C, also modulate cellular signaling pathways and have been implicated in inducing apoptosis [15]. The genomic and structural organization of EVs reflect their evolutionary adaptation to evade host defenses and establish infection across a wide range of tissues. This intricate architecture and replication strategy offer several conserved viral components, particularly in the capsid and NS regions, as attractive targets for the development of antiviral therapies.

### 2.2. Life Cycle of Enteroviruses

EVs follow a conserved replication cycle that includes attachment, entry, uncoating, translation, replication, assembly, and release. The infection begins when EVs attach to host cell surface receptors, many of which are members of the immunoglobulin (Ig) superfamily or integrin receptors [16]. The viral capsid has a “canyon” structure that accommodates the apical domain of Ig-like receptors, triggering conformational changes necessary for uncoating [9,17]. In some enteroviruses, including EV-A71 and CV-A16, which have shallower canyons, receptor binding occurs at regions outside of the canyon, specifically, at the VP1 GH and VP2 EF loops [18,19].

In general, receptor binding is also facilitated by cell surface attachment factors such as heparan sulfate or sialic acid, which enhance virus attachment without necessarily mediating entry or uncoating. Once bound, EVs are typically internalized via clathrin-mediated endocytosis, though alternative endocytic pathways such as caveolin-mediated uptake or macropinocytosis may also be employed, depending on the virus and cell type. After endocytosis, acidification of the endosome promotes conformational changes in the capsid that facilitate genome release into the cytoplasm, usually via pore formation or capsid destabilization. There is also evidence suggesting that some EVs may bypass the endosome and release their genome directly at the plasma membrane [20].

The released positive-sense single-stranded RNA genome is directly translated by host ribosomes to produce a large polyprotein, which is then proteolytically cleaved into structural and NS proteins. The structural proteins, VP1 through VP4, assemble into an icosahedral capsid in which VP1, VP2, and VP3 form the external shell, while VP4 lines the internal surface and associates with the viral RNA [21]. VP4 also undergoes myristoylation, a post-translational modification critical for virion assembly and infectivity [22]. NS proteins encoded by the polyprotein, including 2A–2C and 3A–3D, play key roles in RNA replication, host cell remodeling, and immune evasion [1]. For instance, 2B acts as a viroporin that alters membrane permeability and intracellular calcium homeostasis [23], while 2C, an ATPase and RNA-binding protein, is involved in membrane remodeling for replication complex formation and may contribute to genome packaging [24]. The viral proteases 2A and 3C, as well as their precursor 3CD, mediate polyprotein processing and inhibit host transcription and translation to favor viral protein synthesis [15]. Genome replication is driven by the RNA-dependent RNA polymerase 3D^pol^, which synthesizes a negative-strand intermediate used to generate new positive-sense genomes. This process is primed by the uridylylated VPg protein [25].

Replication occurs within virus-induced membranous compartments derived from host organelles, which concentrate viral components and shield RNA from host defenses. Encapsidation of progeny genomes occurs concurrently with capsid assembly, and virion maturation is facilitated by proteolytic cleavage events and environmental factors such as low pH. Virus release typically occurs via cell lysis, though some EVs can exit through non-lytic, vesicle-associated secretion pathways that may aid in immune evasion and viral spread [13]. Throughout this cycle, EVs manipulate host cellular processes, including gene expression, membrane trafficking, and immune signaling, to create a favorable environment for replication [26]. Despite strain-specific complexities in receptor usage and entry mechanisms, the core steps of the EV life cycle are conserved, offering opportunities for broad-spectrum antiviral targeting.

### 2.3. Clinical Manifestations

Most nonpolio enterovirus infections tend to resolve on their own and are typically asymptomatic, mild, and short-lived. However, in individuals with weakened immune systems—such as infants, young children, or immunocompromised persons—the infection can escalate into serious neurological conditions. EV-A71, CV-A16, and, more recently, CV-A6 are recognized as the primary causes of HFMD, a condition commonly affecting neonates and infants. HFMD is marked by fever, maculopapular or erythematous rashes on the limbs, and painful ulcers in the mouth. Due to their pronounced neurotropism, especially in the case of EV-A71, complications like brainstem encephalitis, acute flaccid paralysis, and aseptic meningitis can arise during seasonal outbreaks. In more severe cases, the viruses may spread to other organs, potentially leading to life-threatening outcomes such as pulmonary edema, septic shock in newborns, or heart dysfunction [27].

Unlike other enteroviruses, EV-D68 infections present differently. These viruses are sensitive to acidic environments and thrive in cooler temperatures, making them more suited to infecting the nasal passages of the upper respiratory tract rather than the acidic gastrointestinal tract. EV-D68 is primarily linked to moderate to severe respiratory syndromes, including severe bronchitis and interstitial pneumonia. Nevertheless, similar to other human enteroviruses, EV-D68 can replicate in neurological tissues and has been classified as a high-risk pathogen, especially following a series of outbreaks in 2014 [28,29].

## 3. Direct-Acting Antivirals (DAAs)

**Figure 1:**
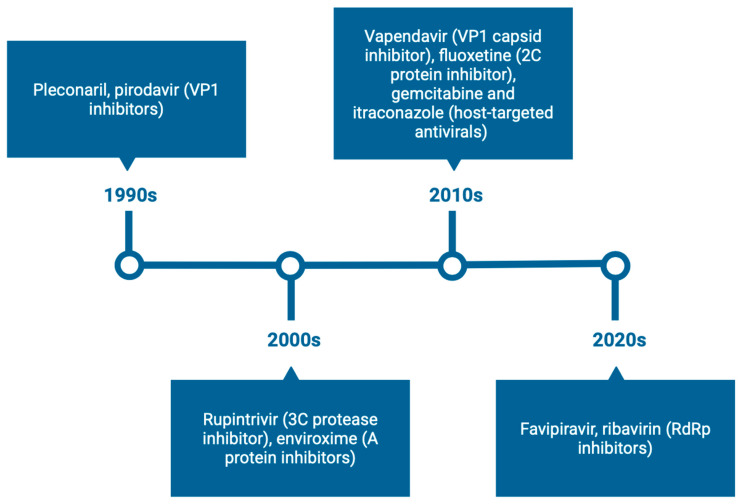
Timeline of key anti-enteroviral compounds and their development (1990s–2020s). Compounds are categorized by target class, including capsid binders (VP1), protease inhibitors (3C), replication complex-targeting agents (3A, 2C, RdRp), and host-targeted or repurposed drugs. This timeline highlights the evolution of direct-acting and host-targeted antiviral strategies against EVs over the past three decades. The figure was created using Biorender.com.

Over the past three decades, various antivirals have been developed to target EVs, including capsid binders, protease inhibitors, replication complex-targeting agents, and host-targeted or repurposed drugs. Figure 1 illustrates the key compounds, their discovery timeline, and current development status, highlighting the evolution of direct-acting antiviral strategies. Early DAAs focused primarily on capsid inhibitors such as pleconaril and pirodavir, followed by protease and replication-targeting agents, with recent efforts exploring repurposed drugs and host-targeted approaches.

### 3.1. Capsid Binders

EVs possess a conserved icosahedral capsid, which is made up of 60 identical protomers, each consisting of four structural proteins: VP1, VP2, VP3, and VP4. The capsid surface is formed by VP1–VP3, while VP4 resides internally [1]. Distinct structural features include star-shaped protrusions formed by VP1 pentamers, circular depressions known as canyons created by VP1–VP3 at the fivefold symmetry axis, and additional features such as the VP2 loop, VP3 knob, and VP1 hydrophobic pocket, typically occupied by a lipid-like “pocket factor” that stabilizes the virion [17]. These features contribute to the antigenic diversity of EVs and offer multiple targets for antiviral drug development. Specifically, three key regions of the capsid have been identified as druggable sites: the VP1 hydrophobic pocket, the VP1–VP3 interprotomer interface, and the five-fold axis responsible for host receptor attachment [30].

Capsid binders represent a broad class of antiviral agents that act by targeting viral surface proteins, thereby preventing viral entry into host cells. Although these compounds are associated with an increased risk of inducing antiviral resistance, they remain a major focus of antiviral research [7]. Among them, pyridyl imidazolidinone and its derivatives have demonstrated potent and selective activity against EV-A71, likely by interacting with the VP1 capsid protein [31]. Rosmarinic acid, another promising candidate, also targets a conserved residue within VP1 and has shown efficacy in both in vitro cell culture and in vivo mouse models [32,33]. In efforts to address the lack of selective antivirals for EV-D68, a novel triazole-based compound was identified that disrupts viral uncoating by binding to the hydrophobic pocket of the VP1 protein [34]. Pleconaril, one of the most studied capsid-targeting antivirals for picornaviruses, has not been approved for widespread use due to safety concerns [35].

As an alternative approach, receptor-blocking antibodies—such as those targeting human scavenger receptor Class B member 2 (SCARB2)—offer another promising strategy by preventing the interaction between viral capsid proteins and host cell receptors [36]. Additionally, some compounds target the positively charged fivefold axis to inhibit receptor attachment; however, efficacy can be strain-dependent. For instance, suramin showed broad activity against several EVs, but was largely ineffective against EV-B members, except for CV-A9 [37]. In addition to well-characterized capsid inhibitors that bind to defined structural pockets, several compounds have demonstrated antiviral activity through interactions with VP1 or the capsid surface, although their precise binding modes remain less clear. For example, lactoferrin and nickel ion/chitosan (NIC) microcomposites are thought to interfere with viral attachment and entry by binding to VP1, while also modulating host immune responses or uncoating [13]. More recently, natural polyphenols like epigallocatechin gallate and resveratrol have been reported to exert antiviral effects by simultaneously binding multiple capsid sites, leading to reduced host cell attachment, virion aggregation, and inhibition of viral RNA release [38]. Together, these findings identified the capsid as a critical and versatile target for the development of broad-spectrum or strain-specific antivirals against EVs.

#### 3.1.1. Antivirals Targeting the VP1 Hydrophobic Pocket

**Figure 2:**
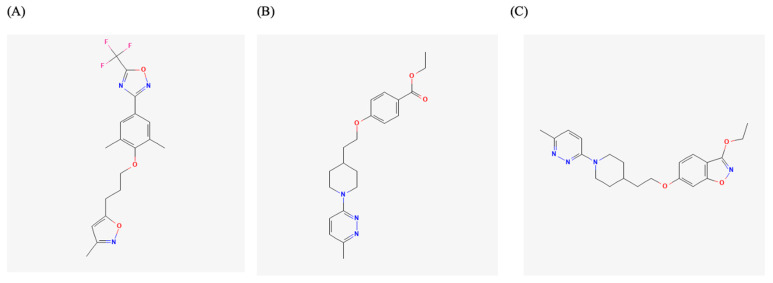
Chemical structures of capsid-binding inhibitors targeting the VP1 hydrophobic pocket of EVs. (**A**) Pleconaril, the prototypical capsid inhibitor widely studied in EV infections (PubChem CID: 1684). (**B**) Pirodavir, a VP1 pocket binder evaluated in clinical trials (PubChem CID: 71345). (**C**) Vapendavir (BTA798), a pleconaril analog with enhanced pharmacological properties (PubChem CID: 504457).

The VP1 hydrophobic pocket is a key target for anti-enteroviral compounds and plays a role in generating variant strains and new EVs. Sequence analyses of over 1600 VP1 variants have revealed conserved residues in the canyon and hydrophobic pocket regions, underscoring the potential of this site as a broad-spectrum antiviral target [39,40]. This capsid region has been extensively investigated in EV-A71, a major cause of HFMD, for the development of antiviral agents. The strategy typically involves replacing the native pocket factor with synthetic compounds that bind more tightly, thereby stabilizing the capsid and preventing viral uncoating. Structural studies, such as X-ray diffraction of WIN 51711 in complex with EV-A71 (PDB: 3ZFE), have helped in defining the binding mode of such capsid binders [41]. Pleconaril, a well-known broad-spectrum WIN compound, initially showed protective effects in EV-A71-infected mice and modest in vitro efficacy (EC_50_ = 0.13–0.54 μg/mL on RD cells), but failed to inhibit certain EV-A71 strains, such as the 1998 Taiwan isolates, due to VP1 mutations that conferred resistance (Figure 2A) [42,43]. In contrast, other capsid binders such as vapendavir and pirodavir displayed better in vitro activity against a range of EV-A71 genogroups, with EC_50_ values between 0.361 and 0.957 µM, though vapendavir’s clinical use was halted due to side effects (Figure 2B,C) [10,44]. More potent inhibitors have since been developed. For instance, a series of benzothiophene derivatives originally developed against human RV A and B also demonstrated activity against EV-A71. Among them, compound VP1-6g showed remarkable potency with an EC_50_ of 15 nM. Time-of-addition and resistance selection experiments suggested that VP1-6g binds to the same pocket on the VP1 protein as pleconaril [45]. PR66, an imidazolidinone derivative, inhibited multiple EV-A71 strains at nanomolar concentrations (IC_50_ ≈ 0.019 µM) and improved survival in mouse models [46]. Related compounds NLD and ALD showed even stronger activity, with IC_50_ values as low as 0.025 and 0.0085 µM, respectively [47]. NLD-22, a derivative with improved selectivity and pharmacokinetics, conferred full protection in mice and bound the VP1 pocket as confirmed by structural studies [48]. Another notable compound, BPROZ-101, a pyridine imidazolidinone derived from WIN compounds, demonstrated exceptional potency (IC_50_ = 0.001 µM) against EV-A71 without significant cytotoxicity. It also exhibited broad-spectrum activity against multiple CV-A (A9, A10, A16, A24), CV-B (B1, B4, B5), and echovirus (E9 and E29) serotypes, though it was ineffective against EV-D68 and some CV-B strains [31,49]. Other compounds like VP1-14 and VP1-15 also demonstrated submicromolar EC_50_ values and in vivo efficacy [50,51]. Broad-spectrum inhibitors such as ICA135, though less potent (EC_50_ in the micromolar range), showed antiviral activity against multiple enterovirus species, including EV-A71 and CV-A16 [18]. Natural products such as auraptene and avoenin have also been reported to inhibit EV-A71, albeit with lower potency and unclear mechanisms [52,53]. Collectively, these findings demonstrated the therapeutic potential of VP1-targeting capsid inhibitors across different EV species, while also highlighting the need to address strain-specific resistance and optimize pharmacological properties for clinical translation.

**Figure 3:**
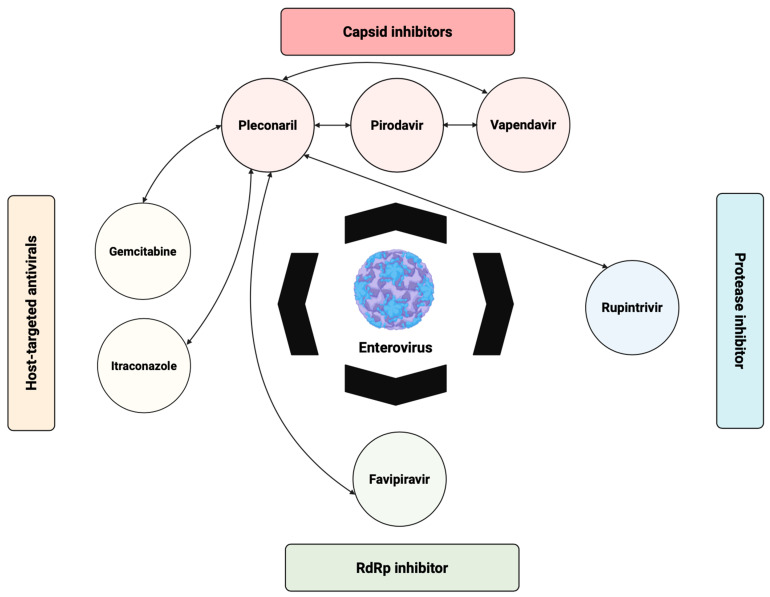
Key investigated anti-enteroviral drug combinations. Major antiviral compounds are grouped in colored circles according to their target class: capsid inhibitors (pleconaril, vapendavir, pirodavir), protease inhibitors (rupintrivir), polymerase inhibitors (favipiravir), and host-targeted antivirals (gemcitabine, itraconazole). Lines indicate experimentally tested combinations that demonstrated additive or synergistic antiviral effects in vitro. This network highlights multi-target strategies aimed at enhancing efficacy, preventing resistance, and providing broad-spectrum coverage across enterovirus species. The figure was created using Biorender.com.

While single-agent capsid inhibitors have shown promising activity against EV-A71 and other EVs, their efficacy can be limited by strain-specific resistance and incomplete coverage across different EV species. To overcome these limitations, combination antiviral strategies targeting multiple stages of the viral life cycle have been investigated (Figure 3). As illustrated in Figure 3, pleconaril, vapendavir, and pirodavir have been tested in combination with other capsid inhibitors, polymerase inhibitors such as favipiravir, protease inhibitors like rupintrivir, and host-targeted antivirals including gemcitabine and itraconazole. Several of these combinations demonstrated additive or synergistic effects in vitro, highlighting the potential of multi-target approaches to enhance efficacy and reduce the emergence of resistance. Notably, combinations of drugs with complementary mechanisms of action appear more promising than combinations of multiple capsid inhibitors, which may offer limited additional benefit due to overlapping targets.

Similar efforts have been directed toward EV-B species, particularly coxsackieviruses and echoviruses, which are associated with severe diseases such as myocarditis and meningitis. Although compounds such as pleconaril and vapendavir have demonstrated promising in vitro inhibition of EV-B infectivity, with IC_50_ or EC_50_ values in the micromolar range, both failed in clinical trials due to limited efficacy and adverse side effects. Nevertheless, their development spurred interest in the design of more effective analogs [54,55]. Most analogs tested showed protective effects in cell-based echovirus assays, with EC_50_ values ranging from 0.3 to 108 µM and selectivity indexes (SIs) between 3 and 1524. Structure–activity relationship (SAR) studies highlighted that the presence of a sulfur atom contributed to activity against CV-B3, while an amino group in the aliphatic chain enhanced anti-echovirus properties [55]. Makarov et al. (2015) developed pyrazolo [3,4-d]pyrimidines active against pleconaril-resistant CV-B3 strains. Compound **36** exhibited favorable antiviral, pharmacokinetic, and physicochemical properties and effectively reduced inflammation and tissue damage in a mouse model of CV-B3-induced myocarditis. In contrast, compound **25** was nearly inactive in vivo due to metabolic inactivation. Compound **36** demonstrated broad-spectrum activity and oral bioavailability, making it a strong drug candidate [56]. Further work by Egorova et al. (2020) involved modifying the pleconaril structure. Alterations at position three of the isoxazole ring led to compounds with improved activity, especially compound **10g**, which was highly effective against both pleconaril-sensitive and -resistant EV strains, with IC_50_ values as low as 0.01 µM. It also showed high oral bioavailability and lacked mutagenic effects, suggesting potential for further development [55]. Carta et al. (2018) identified quinoxaline derivatives with selective and potent activity against CV-B5, a strain associated with myocarditis and encephalitis. Compounds **6** and **7** showed EC_50_ values of 0.09 and 0.06 µM, respectively, along with high SIs. These compounds interfered with early stages of viral infection, likely by blocking internalization rather than virus attachment. Molecular dynamics simulations suggest VP1 as a probable target, although experimental confirmation is needed [57]. When tested against a broader panel of enteroviruses, compounds **6** and **7** maintained activity against CV-B3 and CV-B4 but were inactive against other species. This selective activity may be attributed to variations in the VP1 protein sequences. Additionally, analysis using the RNA-binding protein database suggested that only CV-B3 and CV-B4 share a binding motif for the splicing factor SRSF13, which could represent a novel therapeutic target [58].

EV-D68, a re-emerging pathogen causing acute respiratory illness, which can progress to acute flaccid myelitis, has also been the focus of capsid inhibitor development, although with more limited success. The VP1 hydrophobic pocket of EV-D68 is essential for viral attachment, entry, and uncoating, making it an attractive target for antiviral development. Like other enteroviruses, EV-D68 uses a pocket factor within this conserved site to stabilize the capsid. Capsid-binding antivirals displace this factor, locking the capsid in a rigid state that prevents the conformational changes necessary for host interaction and genome release [59]. Classical WIN-like compounds such as pleconaril, pocapavir, pirodavir, and vapendavir bind to this pocket and exhibit varying degrees of antiviral activity. Pleconaril, originally designed to inhibit EVs and RVs, is particularly effective against EV-D68, with an EC_50_ of 430 nM. This efficacy is attributed to structural similarities between EV-D68 and human RVs, especially the size and position of the pocket factor. Moreover, VP1 residues that interact with pleconaril are highly conserved across over 180 EV-D68 strains spanning several decades, suggesting broad inhibitory potential [60]. However, pleconaril’s clinical development was halted due to inconsistent efficacy and adverse effects [61]. Pocapavir and vapendavir, while effective in poliovirus and HRV-related indications, respectively, failed to inhibit key EV-D68 strains from the 2014 outbreak and the prototype Fermon strain, indicating widespread resistance within this species [62]. In contrast, a tetrazole-based compound, R856932, demonstrated potent activity by targeting the VP1 protein to block viral uncoating, with EC_50_ values ranging from 0.46 to 4.36 μM across contemporary strains. Resistance was linked to mutations in VP1 (A129V) and VP2 (T139A), which affected drug binding and entry. Molecular studies revealed that R856932 shares a binding site with pleconaril, reinforcing its mechanism of action and identifying potential resistance pathways [34]. Recent efforts have also identified quinoline derivatives as promising capsid inhibitors. Structure-based virtual screening and SAR studies led to the development of compound **19**, a quinoline analog containing a 1,2,4-oxadiazole moiety that enhanced its binding affinity to the VP1 hydrophobic pocket. Compound **19** showed potent in vitro activity against both prototype and contemporary EV-D68 strains, along with favorable pharmacokinetics and metabolic stability in preclinical models [63]. These findings highlighted the potential of VP1-targeting antivirals for EV-D68 and support further development of next-generation capsid inhibitors with improved potency and safety profiles.

#### 3.1.2. Antivirals Targeting the VP1-VP3 Interprotomer Binding Pocket

In addition to the classical VP1 hydrophobic pocket, recent research has uncovered an alternative druggable site at the VP1–VP3 interprotomer interface, offering a new avenue for capsid-targeting antivirals, particularly in the EV-B group. Ma et al. (2017) discovered a novel, conserved site on the VP1 protein of CV-B, located near the canyon, but distinct from the hydrophobic pocket targeted by classical capsid inhibitors like pleconaril. This site was identified through a screen of substituted benzoic acids that were highly selective for CV-B viruses and inactive against other EVs, such as echovirus. Among the tested compounds, 4-dimethylamino benzoic acid (4EDMAB) selectively inhibited CV-B3 replication (EC_50_ = 9.4 μM). SAR studies of 23 analogs revealed that adding a hydroxyl group at the ortho position (compound **1**) significantly enhanced potency (EC_50_ = 2.6 μM), while a chlorine substitution (compound **2**) moderately improved activity (EC_50_ = 6.2 μM). However, most modifications to the dimethylamino group were detrimental, except for compound **7**. Notably, the carboxyl group of 4EDMAB interacted with the Arg219 residue, and esterification of this group eliminated antiviral activity [64]. In 2019, Abdelnabi et al. reported a benzene sulfonamide derivative (compound **17**) with potent activity against CV-B3 (EC_50_ = 0.7 μM) and no cytotoxicity at concentrations up to 296 μM. This compound also showed efficacy against CV-B1, B4, B5, and B6, and moderate activity against CV-A9. Notably, further evaluation revealed that this compound and its analogs exhibited broader-spectrum antiviral activity, effectively inhibiting viruses from the EV-C and EV-D groups, as well as RV species A and B. Structural studies revealed that it was binding at an interprotomer pocket formed by two VP1 and one VP3 subunits, a site conserved across EV-B viruses. Although the precise inhibition mechanism remained unclear, it is hypothesized that binding stabilized the virion, increasing the energy barrier for uncoating [9]. Further development of this scaffold led to the discovery of compounds 4 and 7a, both active in the low micromolar range (IC_50_ = 4.29 and 4.22 μM, respectively). SAR analysis indicated that while the phthalimide region could be modified, the carboxyl group was critical for antiviral activity, suggesting its key role in ligand–receptor binding [65]. While most studies to date have focused on the EV-B group, the expanded activity of compound **17** and its analogs against EV-C, EV-D, and RVs suggested that the VP1–VP3 interprotomer pocket might be conserved and exploitable across a broader range of EVs, warranting further investigation into its therapeutic potential beyond EV-B species.

#### 3.1.3. Antivirals Targeting the Five-Fold Axis of the Capsid

In addition to the VP1 hydrophobic pocket and the VP1–VP3 interprotomer site, the five-fold axis of the viral capsid has emerged as another promising target for small-molecule antivirals, particularly in the context of EV-A71. Several compounds have been developed to exploit this conserved and accessible region on the viral surface. MADL385, a dendrimer composed of 12 tryptophan residues, showed antiviral activity against the EV-A71 BrCr strain in vitro with an EC_50_ of 0.28 mM. Cryo-electron microscopy revealed that MADL385 was binding at the five-fold axis of the viral capsid. Drug resistance studies involving mutations at VP1-S184T and VP1-P246S supported this binding mode [66]. Simplified dendrimers with three or four tryptophan residues were subsequently developed to target the same site. One of the most potent compounds, CB-30, inhibited multiple EV-A71 strains with EC_50_ values ranging from 0.2 to 353 nM. Resistance mutations in the same VP1 positions suggested that CB-30 shared the same binding site as MADL385. Structural modeling further confirmed its potential fit around the five-fold axis [67]. Rosmarinic acid, a natural compound, inhibited various EV-A71 strains in vitro with EC_50_ values ranging from 31.57 to 114 μM, but was ineffective against EV-D68. A VP1-N104K mutation conferred resistance and was linked to viral attachment via heparan sulfate. Despite its weaker in vitro potency, rosmarinic acid showed significant in vivo efficacy in an EV-A71-infected mouse model, possibly due to additional mechanisms [32]. Suramin, an FDA-approved antiparasitic drug, inhibited EV-A71 by blocking viral entry. Its antiviral activity was demonstrated in both mouse and monkey models. Although the exact mechanism remained unclear, suramin is believed to bind to a positively charged region around the five-fold axis. However, concerns remain about its broad activity and non-specific binding, as it is a known pan-assay interference compound [37,68]. Suramin analogs, including NF449, NF110, and NM16, also blocked viral entry by preventing EV-A71 attachment to sulfated host cell receptors like heparan sulfate and PSGL-1. Mutations near the five-fold vertex, such as E98Q and K244R, reduced drug sensitivity, confirming the site of action [69]. Brilliant Black BN (E151), a sulfonated azo food dye, inhibited replication of EV-A71, CV-A16, and CV-A6 in cell culture by binding to the five-fold vertex and preventing viral attachment. In vivo, E151 provided complete protection in EV-A71-infected mice and significantly reduced viral loads in the brain and muscle [70]. Overall, the viral capsid, particularly the five-fold axis, is a validated target for EV-A71 antiviral development. While capsid inhibitors offer high potency (in the nano- to picomolar range), they typically have a low barrier to resistance and a narrow antiviral spectrum. To date, no broad-spectrum capsid inhibitors have been reported [10,12]. Despite the promising findings, these five-fold axis-targeting antivirals have so far only been described for EV-A71 and closely related EV-A species. To date, no capsid inhibitors acting through this mechanism have been identified for other EV groups, such as EV-B, EV-C, or EV-D, underscoring a gap in current antiviral discovery efforts. Future research should focus on improving pharmacokinetic properties, enhancing selectivity, and expanding antiviral coverage, potentially through combination therapy strategies. Table 1 summarizes the key capsid-targeting DAAs against EVs, including their mechanisms, spectrum of activity, and representative compounds.

### 3.2. Non-Structural Protein Inhibitors

NS proteins of EVs play essential roles in viral replication and are attractive targets for antiviral drug development. While extensive research has focused on well-characterized targets such as 2C, 3C protease, and 3D RNA-dependent RNA polymerase (3D^pol^), which have shown strong potential for therapeutic intervention, other NS proteins—including 2A^pro^, 2B, 3A, and 3B—remain underexplored. Despite their critical involvement in the viral life cycle, limited literature exists on these proteins, highlighting the need for further investigations into their potential as antiviral targets [7].

#### 3.2.1. 2A Protease Inhibitors

The enterovirus 2A protease (2A^pro^) is a cysteine protease that cleaves the viral polyprotein at the P1/P2 junction and also contributes to processing between 3C and 3D, complementing the activity of 3C^pro^ [13]. Beyond its role in viral polyprotein maturation, 2A^pro^ suppresses host immune responses by cleaving eIF4G to inhibit cap-dependent mRNA translation and hydrolyzing NLRP3 to interfere with IFN-mediated antiviral signaling [71,72]. Despite its central role, 2A^pro^—especially in EV-A71—remains an underexplored drug target, with no potent or highly specific inhibitors being reported.

Early studies have identified weak inhibitors such as Z-LVLQTM-FMK and CW33, which reduce EV-A71 replication only at high concentrations, limiting their therapeutic potential [73,74]. Other compounds, including chlorogenic acid and 1-acetyllycorine, have demonstrated some antiviral effects, though their mechanisms of action remain unclear or lack specificity [75,76]. Natural extracts from *Schizonepeta tenuifolia* and *Melissa officinalis* have shown multitargeted inhibition by disrupting 2A^pro^-mediated cleavage of host factors like eIF4G and hnRNP A1 [77,78]. Moreover, structure-based optimization of 3-benzylcoumarins and repurposed drugs like Telaprevir (against EV-D68) further highlighted emerging efforts in targeting 2A^pro^ [79,80].

Notably, the catalytic triad (His-Asp-Cys) and residues like Ser/Thr125 are highly conserved among enteroviruses, making 2A^pro^ a promising candidate for broad-spectrum antiviral development [81]. The resolved crystal structures of the EV-A71 2A^pro^ mutant (PDB: 4FVB) and its substrate complex (PDB: 4FVD) provided a solid foundation for rational drug design and virtual screening [82]. However, broader investigations across EV species are still needed to fully realize the therapeutic potential of 2A^pro^ inhibition.

#### 3.2.2. 2B Protein Inhibitors

When expressed in *Xenopus* oocytes, the EV-A71 2B protein forms a chloride-selective ion channel, suggesting its classification as a viroporin [83,84]. The chloride channel inhibitor 4,4′-diisothiocyano-2,2′-stilbenedisulfonic acid (DIDS) was shown to inhibit EV-A71 replication in rhabdomyosarcoma (RD) cells. However, due to its structural similarity to other broad-spectrum antiviral compounds like suramin and Brilliant Black, the antiviral activity of DIDS might not be solely attributed to blocking the 2B ion channel. Further studies, including resistance selection assays, are needed to clarify its mechanism of action. Additionally, more selective and potent 2B channel blockers could serve as chemical tools for validating 2B as a drug target. Notably, the 3D structure of the EV-A71 2B protein remains unresolved, and it is still unclear whether its chloride channel activity is critical for viral replication [12]. Interestingly, 2B has also been implicated in host cell apoptosis, as it was found to recruit the proapoptotic protein BAX to mitochondria and trigger cell death [85]. To date, 2B inhibitors have only been identified for EV-A71, with no reported inhibitors targeting the 2B protein of other EV-A species or EVs from other groups, highlighting the need for broader antiviral discovery efforts.

#### 3.2.3. 2C Protein Inhibitors

The 2C protein of EVs is a multifunctional NS protein involved in critical stages of the viral life cycle, including uncoating, RNA replication, membrane remodeling, and encapsidation [86]. It also plays a role in modulating the host’s innate immune response during infection. Although the precise mechanisms through which 2C functions in replication and packaging are still not fully understood, its essential roles and high degree of conservation make it an attractive target for antiviral drug development. Structurally, the 2C protein comprises an ATPase domain, a zinc finger motif, and a C-terminal helical domain. The latter mediates oligomerization by interacting with a concave pocket formed between the ATPase and zinc finger domains. Functional studies have shown that this oligomerization is essential for ATPase activity and efficient viral replication. The X-ray crystal structure of a soluble fragment of EV-A71 2C (residues 116–329) has been resolved at 2.5 Å resolution, offering key structural insights that could facilitate rational drug design [12]. Peptide-based inhibitors such as 2CL and B-2CL, designed based on sequences from EV-A and EV-B 2C, respectively, were shown to disrupt oligomerization and effectively protect mice from EV-A71 infection, providing proof-of-concept for structure-guided antiviral design [87,88].

Several structurally diverse small molecules have been developed to target the 2C protein. Fluoxetine, originally developed as an antidepressant, was explored as a potential antiviral and progressed to clinical trials for EV-D68-associated acute flaccid myelitis, but failed to demonstrate clinical efficacy [89,90]. Later studies revealed that the (S)-enantiomer of fluoxetine was more potent than the racemic mixture against CV-B3 and EV-D68, with higher binding affinity to the CV-B3 2C protein. However, neither the racemic mixture nor the individual enantiomers showed activity against EV-A71, indicating that not all 2C inhibitors exhibited broad-spectrum antiviral activity [91]. Structural analogs of fluoxetine, such as compound **12b**, were developed and demonstrated potent activity against multiple enteroviruses, including EV-A71, EV-D68, CV-B3, poliovirus, CV-A24, HRV-A2, and HRV-B14, with EC_50_ values ranging from 0.0029 to 1.39 μM. Unlike fluoxetine, compound **12b** lacked neuroactivity and did not inhibit serotonin, dopamine, or norepinephrine transporters. Resistance mapping and biochemical assays indicated that mutations conferring resistance to compound **12b** were localized to the α2 helix of 2C, near a solvent-accessible tunnel thought to be involved in inhibitor binding [92].

Dibucaine, a local anesthetic, was identified as a CV-B3 inhibitor through drug repurposing efforts, and it also showed activity against EV-A71 and EV-D68 [93]. SAR studies led to analogs such as compound **12a**, which exhibited enhanced antiviral activity and improved selectivity against EV-D68. Although dibucaine’s original analgesic mechanism involved sodium channel inhibition, these analogs were designed to avoid such activity, minimizing potential side effects [94]. Further optimization yielded compound **6aw**, which displayed favorable in vitro pharmacokinetic properties and a broader spectrum of activity, particularly against EV-A71 [95]. Additional studies led to the identification of compound **6i**, which demonstrated in vivo antiviral efficacy in an EV-A71-infected mouse model and synergized with emetine. However, detailed in vitro and in vivo pharmacokinetic profiles for these compounds have not yet been reported [96].

Another compound, R523062, was identified from a high-throughput screen as an EV-D68 inhibitor and also demonstrated modest activity against EV-A71. Resistance studies pinpointed mutations in the 2C protein, particularly I227L, as a potential driver of resistance. Although attempts to generate a recombinant virus carrying only the I227L mutation failed, likely due to loss of viral fitness, binding assays confirmed that this single mutation was sufficient to confer resistance. Despite its moderate potency, R523062’s simple structure makes it an attractive candidate for further optimization [97].

A series of pyrazolopyridine derivatives were also found to inhibit CV-B3 and displayed broad-spectrum antiviral activity against EV-A71, other coxsackieviruses, echoviruses, and poliovirus [98]. One of the most potent compounds, JX040, inhibited EV-A71 with an EC_50_ of 0.5 μM, although pharmacokinetic profiles remain uncharacterized [99]. Follow-up studies identified compound **7d** as a chemical probe, which binds to 2C proteins from EV-D68, EV-A71, and CV-B3 in differential scanning fluorimetry (DSF) assays. Resistance mapping revealed mutations D183V and D323G in EV-D68 2C that reduced drug sensitivity. However, fitness assays showed that these mutations compromised viral replication, suggesting a trade-off between resistance and viral fitness [100]. Several other 2C inhibitors have been discovered through drug repurposing, including guanidine, TBZE-029, HBB, MRL-1237, pirlindole, zuclopenthixol, metrifudil, formoterol, and N6-benzyladenosine [12,30]. Among these, metrifudil and N6-benzyladenosine were active against EV-A71, while others have yet to be evaluated against this virus. TBZE-029, one of the earliest 2C inhibitors, was able to block positive-strand RNA accumulation, and resistance mutations were mapped to residues 224–229 of CV-B3 2C. Time-of-addition studies confirmed that TBZE-029 acted at the RNA replication stage. Cross-resistance with guanidine hydrochloride suggested a shared mechanism of action [101]. In most cases, resistance mutations were mapped to the viral 2C protein, although direct binding studies have not been conducted to confirm target engagement.

The broad-spectrum antiviral activity of 2C inhibitors highlighted their therapeutic potential. Nevertheless, further studies are required to fully elucidate their mechanisms of action. It is particularly intriguing that structurally unrelated compounds all appear to target the same protein, suggesting the presence of a versatile binding interface or multiple functional hotspots within 2C. To date, only the 2C structures from EV-A71, CV-B3, and poliovirus have been elucidated [87,102,103]. There is a pressing need to determine the 2C structures of other clinically relevant enteroviruses, such as EV-D68, CV-A6, CV-A10, and CV-A16, particularly in complex with inhibitors. Such structural insights would be invaluable for rational drug design and understanding resistance mechanisms. Notably, the X-ray crystal structure of CV-B3 2C in a complex with (S)-fluoxetine was recently reported, revealing an allosteric binding site that stabilized the 2C hexamer. Drug-resistant mutations were found within the (S)-fluoxetine binding site as well as in the AGSINA loop (residues 224–229), which is downstream of the Walker C motif and proposed to help maintain an open conformation favorable for drug binding [103]. The successful crystallization of CV-B3 2C with fluoxetine paves the way for future co-crystal structures of 2C from other enteroviruses, which would further advance lead optimization and antiviral development.

In addition to direct 2C inhibition, targeting host factors that interact with 2C represents another promising strategy. Proteins such as TRIM4, exportin2, and ARFGAP1 are host cofactors that facilitate viral replication by interacting with 2C; disruption of these interactions could impair viral replication and may yield therapeutics with a higher barrier to resistance [104]. Beyond 2C, other NS proteins such as 3A and 3AB were involved in the formation of the viral replication complex [105].

#### 3.2.4. 3A Protein Inhibitors

The replication of EVs occurs within specialized membranous compartments known as replication organelles, which are formed through the subversion of host lipid regulatory pathways. Central to this process is the viral 3A protein, which orchestrates the recruitment and organization of host factors, particularly phosphatidylinositol 4-kinase IIIβ (PI4KB), acyl-CoA-binding domain-containing protein 3 (ACBD3), and oxysterol-binding protein (OSBP) [106,107]. The 3A protein directly interacted with ACBD3, facilitating the localization of PI4KB to replication sites [108]. Structural studies of the 3A-ACBD3 complex across various EVs, including EV-A71, Poliovirus type 1 (PV1), and EV-D68, suggested a conserved mechanism of host factor recruitment [105]. However, the complete structural details of the 3A-host protein complex remain unresolved.

Several small-molecule inhibitors have been identified that disrupt 3A-related functions. Enviroxime, a broad-spectrum inhibitor targeting the 3A pathway, was discontinued in clinical development due to limited efficacy and gastrointestinal side effects [109,110]. Other compounds, such as AN-12-H5 and GW5074, exhibited similar mechanisms and cross-resistance profiles, although their structural dissimilarity from enviroxime raised questions about whether they could bind directly to 3A or modulate its interactions indirectly [111,112,113]. Notably, AN-12-H5 also targeted the viral capsid protein VP1, suggesting potential polypharmacological effects [113]. TTP-8307, another compound with activity against CV-B3, acts by inhibiting oxysterol-binding protein (OSBP)-mediated lipid transport [114,115]. Resistance mutations mapped to the 3A protein suggested a functional link, though direct binding to 3A was not confirmed [12]. Similarly, itraconazole (ITZ), identified through drug repurposing screens, inhibited EV-A71 replication and appeared to target the 3A protein, as resistance mutations were localized to 3A residues. Interestingly, ITZ-resistant strains did not show cross-resistance to other 3A-targeting compounds such as posaconazole or GW5074, indicating the possibility of distinct binding sites or indirect modes of action [116]. Extending their spectrum of activity, ITZ and posaconazole showed efficacy against Parechovirus A3 (PeV-A3), a pathogen associated with severe illness in infants. Resistance mapping revealed mutations in both the capsid (VP0, VP1, VP3) and NS (2A, 3A) proteins, reinforcing the idea that these compounds might act on multiple viral and host targets, including OSBP and oxysterol-binding protein-related protein 4 (ORP4) [117].

Despite the identification of numerous structurally diverse 3A-targeting agents, definitive evidence of direct interaction with the 3A protein was lacking. These inhibitors might exert their effects by disrupting 3A-mediated host protein recruitment rather than by binding to 3A itself. Furthermore, none of the known 3A-targeting compounds have advanced to in vivo evaluation, underscoring the need for further mechanistic studies and the development of biochemical assays to validate 3A as a direct antiviral target. Nonetheless, the multifunctional role of 3A in EV replication continued to make it an attractive candidate for therapeutic intervention. Taken together, these findings highlighted the 3A protein as a multifunctional regulator of viral replication and a compelling, yet underexplored, target for the development of broad-spectrum EV antivirals.

#### 3.2.5. 3C Protease Inhibitors

The 3C protease (3C^pro^) is a key enzyme responsible for cleaving the viral polyprotein at eight specific sites, a process essential for viral replication [118]. In addition to its proteolytic function, 3C^pro^ contributes to host shutoff through cleavage of eukaryotic initiation factor 5B (eIF5B), thereby enabling viral RNA translation to proceed under conditions of eukaryotic initiation factor 2 (eIF2) phosphorylation [119]. Moreover, 3Cpro disrupts host innate immunity by cleaving pattern recognition receptors such as toll-like receptors (TLRs), RIG-I-like receptors, and the Nod-like receptor family pyrin domain-containing 3 (NLRP3), as well as IFN signaling components. It also contributed to the induction of programmed cell death, underscoring its role as a multifunctional virulence factor. Notably, 3C^pro^ showed no homology with mammalian proteases and harbors a conserved His40–Glu71–Cys147 catalytic triad, making it an attractive and specific antiviral target [120,121].

The 3C or 3C-like protease exhibited a strong substrate preference for glutamine at the P1 position, which has provided information for the design of peptide-based inhibitors. These inhibitors typically consisted of di-, tri-, or tetra-peptides containing a pyrrolidone moiety at P1, and were conjugated to reactive warheads such as aldehydes, ketoamides, or α,β-unsaturated esters. Novel warheads like 4-iminooxazolidin-2-one and dually activated Michael acceptors have been developed to enhance metabolic stability and selectivity. Compounds such as **4e** and **4g** demonstrated potent EV-A71 inhibition in the submicromolar range and improved stability in human plasma and microsomes. Similarly, compound **30**, which incorporated a dually activated Michael acceptor, showed reduced off-target activity against host proteases such as cathepsin K and calpain I [122,123].

**Figure 4:**
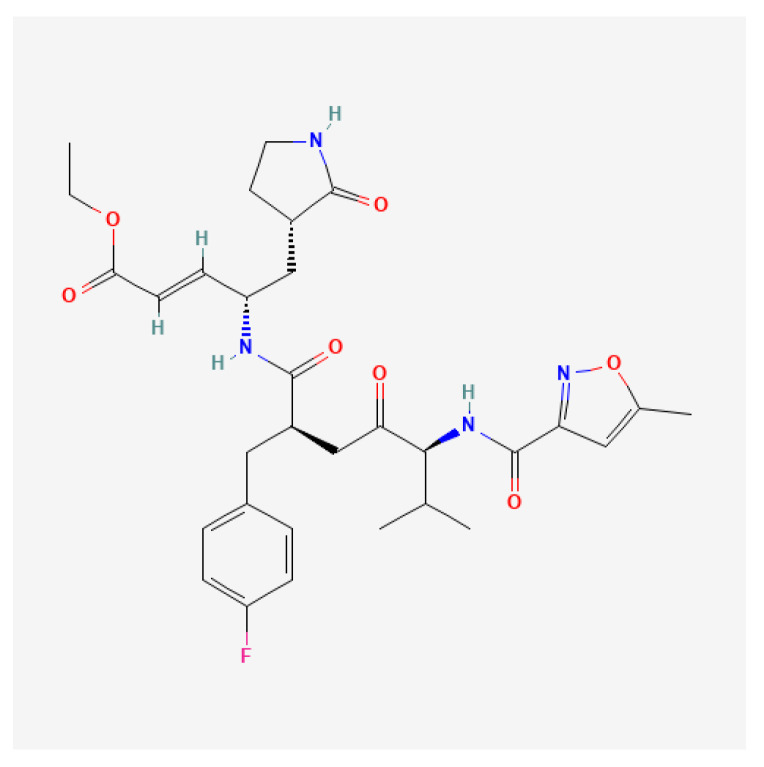
Chemical structure of the 3C protease inhibitor Rupintrivir (AG7088). Rupintrivir is a peptidomimetic inhibitor that targets the viral 3C^pro^, an essential enzyme for enterovirus polyprotein processing. It advanced to Phase II clinical trials, with intranasal formulations tested after oral delivery showed limited efficacy (PubChem CID: 6440352).

Non-covalent inhibitors have also been reported. DC07090, identified through virtual screening, exhibited moderate enzymatic inhibition and displayed antiviral activity, but introduced a new chemotype for future optimization [124]. Rupintrivir (AG7088), a selective, irreversible 3Cpro inhibitor originally developed by Pfizer to treat the common cold, was advanced to clinical trials for RV infections, but failed to show clinical efficacy in naturally infected patients due to poor oral bioavailability (Figure 4) [125,126]. AG7404, a close analog with a rigid pyridine ring replacing the isopropyl side chain of rupintrivir, was developed to improve oral bioavailability. It exhibited favorable pharmacokinetics and safety in Phase I trials, making it a promising candidate for further development [126,127]. Interestingly, the combination of rupintrivir and pleconaril showed a synergistic antiviral effect against EV1 in vitro, suggesting the potential for combination therapy strategies [128]. Rupintrivir was also shown to inhibit a broad range of EVs, including EV-A71, CV-A16, EV-D68, and noroviruses, though it lacked activity against severe acute respiratory syndrome coronavirus 2 (SARS-CoV-2) [118,129,130]. Structural studies revealed covalent binding of rupintrivir to the catalytic cysteine (C147) of EV-A71 3C^pro^ [118,131]. However, the semi-closed S2 pocket and smaller S1’ subunit of EV-A71 3Cpro reduced binding affinity to rupintrivir, limiting its efficacy [118]. Compound **12**, with the 5-methylisoxazole ring of rupintrivir being replaced with a trifluoromethyl group, showed potent activity against echovirus 7 with an EC_50_ of 65 nM, which was comparable to rupintrivir’s efficacy against EV-A71 [132].

Fragment-based screening has identified additional irreversible inhibitors, such as compound **7a**, effective against CV-B3 and EV-D68 3C^pro^, though their activity against EV-A71 remained untested [133]. Other compounds like GC373, GC375, and GC376 have shown antiviral activity in vitro [129]. NK-1.8k is a potent 3C^pro^ inhibitor with low micromolar activity against various EV-A71 strains [134]. However, its reduced potency in Vero cells might be attributed to P-glycoprotein (P-gp)-mediated efflux, which could be mitigated by co-treatment with a P-gp inhibitor [130,135]. Resistance studies identified the N69S mutation in 3C^pro^, which diminished protease activity and reduced NK-1.8k binding, as confirmed by crystallographic analysis. Its analog, NK-1.9k, demonstrated even more potent antiviral activity (EC_50_ = 24.9 nM) and structural studies showed covalent interaction with the catalytic site (PDB: 5GSO) [136].

Efforts to develop broad-spectrum antivirals led to the discovery of α-ketoamide-based inhibitors such as compounds 11r and 18p. The latter displayed potent antiviral activity against EV-A71 and other EVs such as EV-D68, CV-A21, CV-B3, RV-B14, and RV-A02-WT, as well as SARS-CoV-2, despite modest enzymatic inhibition. X-ray structures confirmed the formation of a covalent bond with the catalytic cysteine. Compound **18p** (PDB: 7DNC) also exhibited a long half-life of 5.85 h following intravenous administration in mice, suggesting favorable pharmacokinetic properties for in vivo studies [137]. Structure-guided development of α-ketoamides identified compound **13** as a dual inhibitor of EV and coronavirus proteases, with a covalent bond formed between the α-ketoamide carbon and catalytic cysteine. Subsequent optimization of the P2 substituent led to compounds 14 and 15, which demonstrated improved activity in Vero cells and favorable safety profiles [137]. Switching the α-ketoamide warhead to an aldehyde resulted in compound **16**, which showed potent activity against EV-D68 and CV-A21, but reduced activity against CV-B3 was likely due to substrate pocket differences [138].

Macrocyclic inhibitors such as compound **4** have also been explored, with structural validation confirming their interaction with 3C^pro^ [139]. Cyanohydrin-based inhibitors have emerged as selective cysteine protease inhibitors. Compound (R)-1, a cyanohydrin derivative, showed potent inhibition of EV-A71 with an EC_50_ of 0.048 µM [140]. Further modifications led to the development of stable isosteres like 4-(4-fluorophenyl)-2-iminooxazolidin-5-one cyanohydrin (FOPMC) and 4-(2-fluoro-4-iodophenyl)-2-iminooxazolidin-5-one cyanohydrin (FIOMC) by replacing the acyl cyanohydrin with 4-iminooxazolidin-2-one, offering a novel scaffold for future inhibitor design [122,141]. Natural products such as luteoloside have also been identified as non-peptidomimetic 3Cpro inhibitors with moderate activity, highlighting alternative strategies for targeting the protease [142]. Additionally, high-throughput virtual screening identified a new class of covalent inhibitors, including compound **18** derived from phenylthiomethyl ketone 17, which showed binding to 3Cpro via reversible or irreversible mechanisms depending on the warhead orientation. These represented novel scaffolds for future optimization [133]. To avoid the potential off-target effects of covalent inhibitors, an allosteric inhibitor, benserazide—a Parkinson’s disease drug—was discovered to noncompetitively inhibit CV-B3 3Cpro. SAR expansion led to analogs with submicromolar IC_50_ values, although some lacked corresponding antiviral activity, likely due to limited cell permeability [143].

Additionally, host proteins like ATG4B have been implicated in viral polyprotein processing. Identified using a rupintrivir-derived probe, ATG4B exhibited protease activity similar to 3C^pro^, and its knockdown impaired EV-A71 replication. However, the exact role of ATG4B in the viral life cycle remained unclear [144]. Despite promising in vitro findings and the broad-spectrum potential of 3C^pro^ inhibitors, in vivo efficacy against EVs has yet to be demonstrated. Furthermore, concerns remain regarding potential off-target effects of covalent inhibitors, particularly their activity against host cysteine proteases such as cathepsin L, calpain 1, and cathepsin K [130,145,146]. Taken together, these advances underscore the therapeutic potential of 3C^pro^ as a high-value antiviral target, and continued structure-based optimization, coupled with in vivo validation, would be critical to translating these promising candidates into clinically effective EV therapeutics.

#### 3.2.6. Three-Dimensional Polymerase Inhibitors

The RNA-dependent RNA polymerase (RdRp), also known as 3D^pol^, plays a central role in the replication of EV genomes by catalyzing the synthesis of both negative- and positive-strand viral RNA. In addition to catalyzing RNA strand synthesis, 3D^pol^ also mediates the uridylation of VPg, a critical step in initiating viral RNA replication [13]. Given its essential and conserved function across EVs, 3D^pol^ represents a highly attractive antiviral target. The majority of reported 3D^pol^ inhibitors are nucleoside or nucleotide analogs that mimic natural substrates and interfere with RNA synthesis by chain termination or by introducing lethal mutations. However, several non-nucleoside inhibitors with distinct mechanisms of action have also emerged.

High-throughput screening of FDA-approved drugs identified gemcitabine, LY2334737, and sofosbuvir as effective nucleoside analogs with anti-EV-A71 activity. Notably, LY2334737 and sofosbuvir not only reduced viral titers in vitro, but also protected mice from lethal EV-A71 challenge, attenuating viral replication, and tissue damage. Gemcitabine exhibited synergistic effects when used in combination with IFN-β, suggesting its potential use in combination therapies [147]. Time-of-addition studies indicated that gemcitabine acted after viral entry, possibly through two mechanisms: incorporation into viral RNA leading to mutations, and direct interference with 3Dpol by preventing nucleotide incorporation.

Gemcitabine also demonstrated synergistic antiviral activity when combined with ribavirin, reducing CV-B3 replication by up to 80%. This combination yielded a combination index (CI) of less than 1, confirming synergism [148]. In addition, gemcitabine suppressed pyrimidine biosynthesis and activated IFN-stimulated genes (ISGs), enhancing innate antiviral immunity [149]. The cytidine analog, 2′-deoxy-2′-β-fluoro-4′-azidocytidine (FNC), originally developed for human immunodeficiency virus (HIV), demonstrated nanomolar-level antiviral activity against multiple EVs, including EV-A71, CV-A16, CV-A6, EV-D68, and CV-B3. FNC was shown to bind to EV-A71 3D^pol^ and inhibit polymerase function, although it remained unclear whether it was incorporated into viral RNA as a chain terminator. In mouse models, FNC significantly reduced viral loads and protected against mortality, indicating its promise as a broad-spectrum antiviral agent currently under phase II clinical evaluation in China [150].

**Figure 5:**
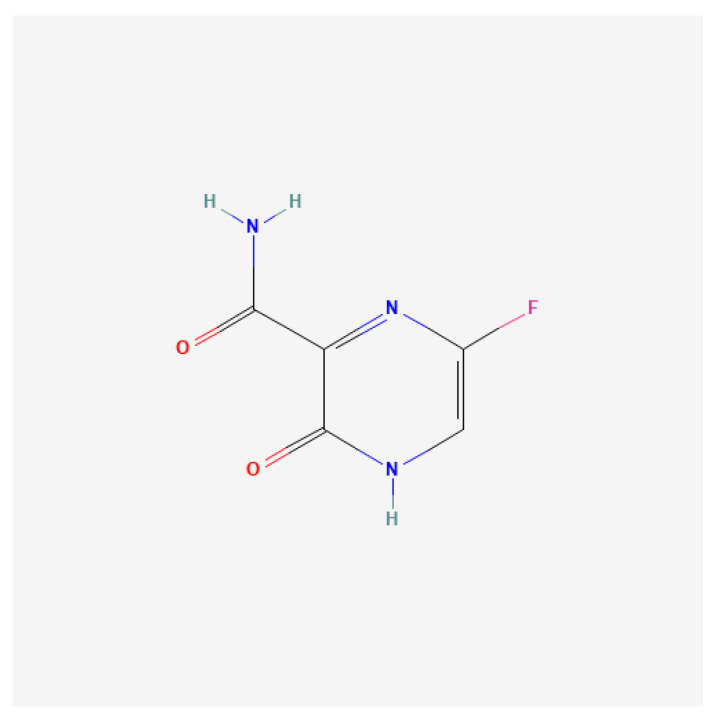
Chemical structure of the RdRp inhibitor Favipiravir (T-705). Favipiravir, an approved anti-influenza drug in Japan, has demonstrated in vitro activity against EVs (PubChem CID: 492405).

Another broad-spectrum antiviral, favipiravir, inhibited EV-A71 replication by targeting the 3D^pol^ (Figure 5). Resistance mapping revealed the S121N mutation in the polymerase domain as the determinant of reduced drug sensitivity, confirming 3D^pol^ as the molecular target [151]. Similarly, the adenosine analog NITD008, initially developed for flaviviruses, inhibited EV-A71 and other enteroviruses with EC_50_ values ranging from 0.1 to 4.9 µM. Its triphosphate form acted as a chain terminator in RdRp assays. Combination therapy using NITD008 with capsid (GPP3) or protease (AG7088) inhibitors produced synergistic effects. In vivo, NITD008 significantly decreased mortality and viral loads in infected mice. However, its development was discontinued due to adverse side effects in clinical trials [152,153,154]. Other nucleoside analogs with anti-EV activity included remdesivir, a broad-spectrum antiviral approved for coronavirus disease 2019 (COVID-19). Remdesivir inhibited EV-A71 replication post-entry and reduced both negative- and positive-strand RNA synthesis. It also displayed efficacy against CV-B3 and EV-D68, with EC_50_ values of 0.097 and 0.026 µM, respectively, highlighting its pan-enterovirus potential [155]. Another nucleoside analog, MRS7704, demonstrated inhibitory activity against EV-A71, with a reported EC_50_ of 3–4 µmol/L [156].

Several non-nucleoside inhibitors of 3D^pol^ have also been characterized. DTriP-22 exhibited submicromolar efficacy against multiple EV-A71 and coxsackievirus strains. Resistance mutations mapped to the 3D^pol^ protein, specifically R163K, as well as in the 2C and VP1 regions. Biochemical assays confirmed that DTriP-22 inhibited poly(U)-elongation by 3D^pol^ without affecting VPg uridylation [157]. Aurintricarboxylic acid also suppressed 3D^pol^ activity in vitro, although resistance studies have not yet been conducted to validate its mechanism [158]. GPC-N114 is among the most potent non-nucleoside 3D^pol^ inhibitors reported, with an EC_50_ of 0.13 µM against EV-A71. Structural studies revealed that GPC-N114 was able to bind to the RNA-binding channel of CV-B3 3D^pol^ and blocked RNA elongation [159]. Crystallographic data showed that one 2-cyano-4-nitrophenyl ring of GPC-N114 was exposed to solvent while the other occupied the nucleotide acceptor pocket, overlapping the template primer binding site. These features contributed to its mechanism of action, which prevented nucleotide incorporation and impaired RNA elongation. Comparative studies with encephalomyocarditis virus (EMCV) and CV-B3 suggested that resistance mutations occurred near the GPC-N114 binding site, supporting its conserved mechanism across picornaviruses [159]. Similarly, BPR-3P0128 inhibited both RdRp activity and VPg uridylation with exceptional potency (EC_50_ = 2.9 nM) against EV-A71. Notably, it retained efficacy against DTriP-22-resistant strains, although no resistance mutations to BPR-3P0128 were identified, suggesting a potentially robust resistance profile [160].

Despite the clear promise of polymerase inhibitors, challenges remained in their development. Nucleoside analogs often carry the risk of host polymerase inhibition, leading to toxicity, and might possess immunomodulatory properties. While non-nucleoside compounds offered alternative mechanisms and potentially improved selectivity, few have progressed to in vivo evaluation or clinical testing. Nevertheless, the high conservation of 3D^pol^ across EVs and the success of polymerase inhibitors in other viral systems affirm its status as a high-risk, high-reward antiviral target. Future studies should focus on structure-guided drug design, resistance profiling, and in vivo validation to fully exploit this critical enzyme for therapeutic development. The non-structural protein inhibitors against EVs, including their mechanisms, spectrum of activity, and representative compounds are presented in Table 2.

## 4. Host-Targeting Antivirals

EVs are obligate intracellular pathogens that depend on host cellular machinery and signaling pathways for successful replication. Targeting these host factors could offer an alternative antiviral strategy, with potential advantages including broad-spectrum activity and a high barrier to resistance. However, concerns about cytotoxicity remained a key limitation of host-targeted therapies.

Various high-throughput approaches, such as siRNA screening, proteomics, and insertional mutagenesis, have been used to identify essential host factors involved in EV-A71 replication [12,163]. One critical pathway is the phosphatidylinositol 4-kinase III beta–phosphatidylinositol 4-phosphate–oxysterol-binding protein (PI4KB–PI4P–OSBP) axis, which is utilized by EVs to deliver cholesterol to replication organelles. PI4KB is essential for forming replication organelles across all EVs, while OSBP binds PI4P and mediates lipid exchange between the endoplasmic reticulum and the replication membrane [164,165]. OSW-1, a natural compound that targets OSBP, exhibited potent antiviral activity against enteroviruses, including EV-A71, with EC_50_ values in the nanomolar range [166,167]. Short-term treatment with OSW-1 (1–6 h) reduced OSBP levels by approximately 90% in various cell lines without noticeable toxicity, and this reduction persisted across cell generations. Although the mechanism underlying this persistent OSBP depletion was not fully understood, OSW-1-treated cells showed resistance to EV infection, suggesting its potential as a prophylactic agent [168].

ITZ also targets OSBP and its homolog ORP4. While some reports indicated ITZ might act on the viral 3A protein, functional studies showed that OSBP knockdown impaired viral replication, and overexpression of OSBP or ORP4 reversed the antiviral effects of ITZ and OSW-1. ITZ was proposed to inhibit the lipid-shuttling activity of OSBP, which is necessary for replication organelle formation [169]. Additional OSBP-targeting antivirals include ITZ analogs such as TTP-8307 and T-00127-HEV2. These compounds modulated the OSBP function through distinct binding modes, and their structural diversities opened up the possibilities for combination therapy. Optimization of ITZ analogs has identified structural features essential for OSBP selectivity, aiming to reduce cytotoxicity associated with ORP4 binding [30,170]. The host kinase, PI4KB, is also a druggable target. MDL-860 was found to irreversibly modify cysteine 646 (C646) in an allosteric pocket distant from the active site, thereby inhibiting PI4KB function. Notably, the C646S mutant retained enzymatic activity, indicating that this allosteric site could be targeted without affecting PI4KB’s essential catalytic functions and potentially reducing off-target effects [171]. Other PI4KB inhibitors included enviroxime-like compounds such as oxoglaucine, TTP-307, PIK93, and T-00127-HEV1, which was shown to inhibit PI4KB and exhibited antiviral activity against multiple coxsackievirus B strains [30]. Compound **10**, a second-generation derivative combining features of PIK93 and T-00127-HEV1, showed potent nanomolar-level activity against CV-B3 in cell-based assays [172]. MDL-860, another enviroxime-like compound, irreversibly inhibited PI4KB through covalent modification at an allosteric cysteine residue (C646), and its analog compound A1 provided 50% protection in CV-B1-infected neonatal mice [173].

RYL-634 is a broad-spectrum antiviral compound identified through phenotypic screening and SAR studies. It was able to inhibit multiple viruses, including EV-A71, with an EC_50_ of 4 nmol/L. Its target was identified as the human dihydroorotate dehydrogenase (DHODH), an enzyme involved in pyrimidine biosynthesis. The antiviral activity of RYL-634 against the hepatitis C virus was reversed by uridine supplementation, supporting the involvement of DHODH [174,175,176]. Another DHODH inhibitor, FA-613, demonstrated broad-spectrum antiviral activity in the low micromolar range against viruses such as the influenza virus, EV-A71, respiratory syncytial virus (RSV), RVs, and coronaviruses [177]. Natural compounds such as aloe-emodin, all-trans-retinoic acid, anisoside B4, and ginsenoside Rb1 also exhibited antiviral activity by inducing type I and III IFNs in infected cells [13]. TLR7 agonists such as R837 showed protective effects in EV-A71-infected mice by activating innate immune responses and preventing paralysis and death [178]. GS-9620, a TLR7 agonist, improved survival and reduced clinical symptoms in EV-A71-infected mice. Its antiviral activity might involve the activation of NF-κB and PI3K pathways. Treatment with GS-9620 reduced levels of inflammatory cytokines such as IFN-α, IFN-γ, and MCP-1 [179]. Additionally, co-treatment of IFN-α with the 3Cpro inhibitor Rupintrivir at a ratio of 1:40 or 1:200 produced a synergistic antiviral effect (combination index: 0.14–0.27) [180]. Intrinsic restriction factors have also emerged as key modulators of EV replication. The host protein sterile alpha motif and histidine–aspartate domain-containing protein 1 (SAMHD1) prevented EV-A71 and CV-A16 replication by disrupting the interactions between viral capsid proteins VP1 and VP2, thereby interfering with viral assembly. This activity was shown to be independent of its dNTPase function [181,182]. However, EV-A71 upregulated tripartite motif-containing protein 21 (TRIM21) to facilitate proteasomal degradation of SAMHD1, countering this antiviral effect [182,183]. Another restriction factor, APOBEC3G (A3G), impaired EV-A71 and EV-D68 replications by binding to the cloverleaf and stem-loop II structures within the 5′ UTR, competing with the host translation factor poly(rC)-binding protein 1 (PCBP1). This disrupted viral RNA translation and replication [184].

Heparan sulfate proteoglycans (HSPGs) function as attachment factors for EV-A71. A library of sulfated heparan sulfate disaccharides was developed as decoy receptors to inhibit virus attachment. Among them, HTA-22, a per-sulfated GlcN-α(1,4)-Glc synthetic HS mimetic, showed the strongest inhibitory activity, with an IC_50_ of 7.9 µmol/L. Time-of-addition experiments confirmed that HTA-22 blocked viral attachment to cells [185,186]. Additional HSPG mimetics, including heparin, heparan sulfate, and pentosan polysulfate, were evaluated, with heparin inhibiting EV-A71 replication by over 90% at 7.81 µg/mL, mainly by interfering with early viral entry [187]. Lactoferrin, particularly from bovine sources, inhibited EV-A71 replication in RD and SK-N-SH neuronal cells. It was able to bind to both the cell surface and viral capsid protein VP1. Administration of lactoferrin improved survival rates in mice infected with EV-A71. Although it was suggested that lactoferrin might block viral binding to HSPGs, direct evidence of this interaction is lacking [188]. Human N-myristoyltransferase 1 (hNMT1), but not hNMT2, was found to be critical for EV-A71 replication, as EV capsid protein VP4 contains a conserved myristoylation motif. Inhibition of hNMT1 using siRNA or the small-molecule inhibitor compound **4O** suppressed viral replication by preventing proper processing of capsid precursors such as VP0, VP4-2-3, and P1 [22]. The host protein AP2M1 was recently identified as a conserved factor interacting with the YxxØ motif found in several viral proteins, including the EV 2C protein. AP2M1 facilitated the localization of 2C to the ER. Inhibition of the AP2M1–YxxØ interaction using N-(p-amylcinnamoyl)anthranilic acid (ACA) disrupted 2C localization and reduced colocalization with the ER from 63% to 21%, resulting in significant inhibition of EV-A71. ACA also showed in vitro and in vivo efficacy against other viruses, such as influenza, Zika virus, and Middle East respiratory syndrome coronavirus (MERS-CoV) [189].

Torin2, an ATP-competitive mTOR kinase inhibitor, showed potent anti-EV-A71 activity with an IC_50_ of 0.01 µmol/L. A derivative compound, HTA-11e, demonstrated even greater potency, with an IC_50_ of 0.027 µmol/L. However, the specific role of mTOR signaling in EV-A71 replication remains to be fully elucidated [190]. Torin2 and related compounds were able to inhibit virus-induced autophagy, a pathway subverted by EV-A71 for replication. The Torin2 derivative LY-55 (4 µM) reduced JNK phosphorylation and inhibited the autophagy marker LC3-II, while enhancing P62 expression. LY-55 acted synergistically with 3-MA, a phosphoinositide 3-kinase (PI3K) inhibitor, to suppress autophagy and viral replication [191]. EV-D68 has also been shown to activate the receptor expressed on myeloid cells 1–nuclear factor kappa-light-chain-enhancer of activated B cells–mitogen-activated protein kinase (TREM-1–NF-κB–MAPK) axis, promoting the release of interleukin (IL)-6, IL-8, and tumor necrosis factor alpha (TNF-α). Pharmacological blockade of TREM-1 using the LP17 peptide reduced NF-κB-mediated transcription and downstream p38 MAPK activation, indicating this inflammatory signaling cascade as a host-targeting strategy with both antiviral and immunomodulatory benefits [192].

**Figure 6:**
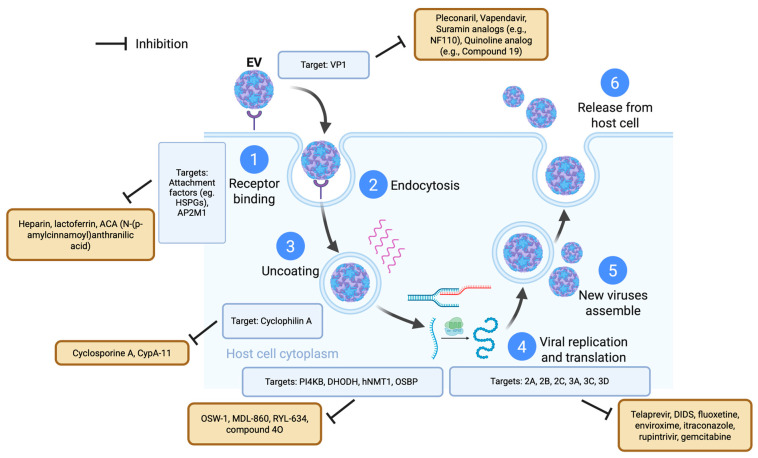
Schematic representation of the EV life cycle highlighting viral and host targets of current antiviral strategies. Key stages of the EV life cycle are illustrated, including viral attachment (e.g., HSPGs), endocytosis and uncoating (e.g., cyclophilin A), viral replication and translation (e.g., PI4KB, OSBP, hNMT1, DHODH), and virion assembly and egress. The roles of structural proteins (e.g., VP1) and non-structural proteins (e.g., 2A, 2B, 2C, 3A, 3B, 3C, 3D) are shown alongside representative inhibitors targeting each component. This figure integrates both host-targeted and direct-acting antiviral approaches across the EV life cycle. The figure was created using Biorender.com.

Cyclophilin A (CypA), a peptidyl-prolyl isomerase, plays a role in the replication of multiple viruses, including EV-A71 [12]. CypA interacted with VP1 and was involved in viral uncoating [193]. Knockdown of CypA via shRNA reduced viral replication. CypA inhibitors such as HL051001P2 and cyclosporine A inhibited EV-A71 with EC_50_ values of 0.78 µmol/L and 3.38 µmol/L, respectively. A lead-optimized derivative, CypA-11, demonstrated even stronger antiviral activity with an EC_50_ of 0.37 µmol/L [194]. CypA-11 also exhibited synergistic effects in the 0.31–5 µM range when combined with the 3Cpro inhibitor NK-1.8K. However, a VP1-S243P mutation conferred resistance to CypA inhibition by disrupting VP1–CypA binding [193,194]. In addition to CypA, host DNA topoisomerase I (TOP1) was implicated in RNA replication and protein synthesis of EV-A71. Although not essential for host cell viability, TOP1 inhibition using camptothecin effectively suppressed EV-A71 replication, highlighting the potential of targeting non-essential host factors as complementary antiviral strategies [161]. Overall, host-targeting antivirals provided valuable insights into virus–host interactions and could offer promising therapeutic avenues. Strategies to reduce off-target effects include focusing on host factors specifically upregulated during infection or combining host-targeted therapies with direct-acting antivirals. Table 3 presents key host-targeting antiviral strategies against EVs, highlighting their molecular targets, mechanisms of action, representative compounds, and reported antiviral effects. Figure 6 illustrates the EV life cycle and maps key viral and host targets of antiviral strategies, including structural (VP1) and non-structural proteins (2A–3D), as well as host factors involved in viral entry, replication, and immune modulation.

## 5. Emerging and Experimental Strategies

### 5.1. Immunotherapeutic Approaches

Human intravenous immunoglobulin (hIVIG), consisting of pooled IgG antibodies from healthy donors, has demonstrated broad neutralizing activity against several EVs, including EV-D68. Studies showed that commercial IVIG preparations contained high titers of neutralizing antibodies, suggesting both shared and unique antigenic determinants when compared to historical EV strains. In animal models, IVIG treatment significantly reduced paralysis incidence and motor deficits, and clinical use in neonates with severe EV infections has led to rapid viral clearance via pathogen-specific antibody responses [59,195].

Monoclonal antibodies (mAbs) could offer a targeted strategy against EVs by binding and neutralizing free viral particles. For EV-D68, multiple mAbs showed protective effects in mice when administered either before or after infection. For instance, antibody EV-D68-228 was able to bind the viral capsid’s five-fold axis, while mAb A61 targeted the VP1 DE loop and prevented interaction with α2,6-linked sialic acid receptors [196,197]. More recently, mAbs 15C5 and 11G1 were found to neutralize EV-D68 through distinct mechanisms, like inducing conformational changes in the viral particle or mimicking receptor interactions to block infection [198].

The innate immune system, particularly type I IFNs, plays a critical role in early antiviral defense. Virus recognition by pattern recognition receptors (PRRs) such as TLR3, TLR7, TLR8, and RIG-I triggers a cascade leading to IFN-β production through IRF3 activation. This initiates a positive feedback loop involving IRF7, resulting in the amplification of IFN-α/β and the expression of ISGs [199]. Several studies have highlighted the importance of IFN signaling in EV infections. For example, mice lacking type I IFN receptors showed significantly reduced survival after CV-A16 infection [180]. Moreover, 3C^pro^ from EV-A71 was shown to degrade IRF9 and impair IFN signaling [200]. Combination therapy with IFN-α and the protease inhibitor, rupintrivir, showed synergistic suppression of EV-A71 replication [180]. Similarly, EV-D68 3C^pro^ targeted IRF7 for degradation, further underscoring how viral proteases could evade innate immunity by disrupting IFN responses [201].

Taken together, immunotherapeutic strategies represented a vital and evolving front in the fight against EV infections. Passive immunization using hIVIG and monoclonal antibodies has demonstrated protective efficacy, particularly in severe cases and in vulnerable populations, while offering insights into viral antigenicity and neutralization mechanisms. Concurrently, bolstering the innate immune response through type I IFN signaling provided a promising avenue to counteract EV-mediated immune evasion. However, the heterogeneity among EV species and their diverse strategies to subvert host immunity underscore the need for targeted, virus-specific interventions. Continued investigation into host–pathogen interactions, coupled with advances in antibody engineering and immune modulation, would be crucial for translating these immunotherapeutic approaches into effective clinical treatments for EV-associated diseases.

### 5.2. RNA Interference (RNAi)

RNAi is a gene-silencing process that occurs after transcription and was first observed in pigmented petunias in 1990 [202]. It was first demonstrated that this potent gene-silencing effect was initiated by double-stranded RNA (dsRNA) in *Caenorhabditis elegans*, leading to the degradation of matching mRNA molecules [203]. The RNAi pathway is driven by Dicer, an RNase III family endonuclease, which generates small RNA molecules that guide the Argonaute (AGO) protein—an essential part of the RNA-induced silencing complex (RISC)—to degrade complementary mRNAs. These small RNAs include microRNAs (miRNAs), derived from hairpin-shaped precursor miRNAs (pre-miRNAs), and small interfering RNAs (siRNAs), processed from long dsRNAs [204].

RNAi is increasingly recognized as a conserved antiviral mechanism in eukaryotes, though its role in mammals has been a subject of debate. This skepticism largely stems from the dominant presence of IFN-mediated responses and adaptive immunity in mammals, which are absent in invertebrates and plants. Some studies suggested a competitive or inhibitory interaction between the RNAi and IFN pathways, with IFN-associated proteins such as LGP2 shown to suppress Dicer, a key enzyme in RNAi [205,206]. Despite this, accumulating evidence supported the existence of functional antiviral RNAi in both undifferentiated and differentiated mammalian cells [207,208]. In mammals, Dicer processes viral dsRNA intermediates into 21–23 nucleotide virus-derived small interfering RNAs (vsiRNAs), which are loaded into AGO2, the only slicing-competent AGO protein, to mediate degradation of complementary viral RNAs [204]. Notably, an alternative isoform of Dicer, termed antiviral Dicer (aviD)*,* was recently discovered. This isoform lacks the Hel2i domain and exhibits enhanced capacity to generate antiviral siRNAs, offering increased protection against RNA viruses such as Zika virus and SARS-CoV-2 [209]. To evade host RNAi responses, many viruses encode viral suppressors of RNAi (VSRs) [210].

CV-B3 has been the most extensively studied EV in the context of RNAi-based antiviral strategies. Early investigations demonstrated that siRNAs targeting conserved regions of the viral 3D polymerase (3D^pol^) effectively reduced CV-B3 replication by 80–90% in vitro, with protective effects lasting several days. Additionally, siRNAs against the host cell receptor CAR significantly reduced viral titers, and their combination with viral-targeting siRNAs was proposed as a robust dual-targeting approach [211]. siRNA-2A targeting the viral 2A protease showed potent antiviral effects in vitro and in a susceptible mouse model, reduced viral titers, tissue damage, and improved survival. However, repeated administrations were required for sustained effects due to siRNA degradation [212]. Another potent siRNA, siRNA-4, directed against the 2A region, showed 92% inhibition in vitro and protected cells both pre- and post-infection. This siRNA displayed strand-specific activity, targeting the positive-sense RNA with high specificity and without inducing escape mutants, likely due to targeting at a highly conserved and essential site [213]. Kim et al. (2007) designed shRNAs targeting several genomic regions, including 3D^pol^ and VP1, with shRNA-2 and shRNA-5 demonstrating in vivo efficacy in reducing viral load and tissue damage in mice [214]. Fechner et al. (2007) demonstrated that stable coxsackievirus–adenovirus receptor (CAR) knockdown via vector-delivered shRNA achieved 97% inhibition of CV-B3 in cardiac cells and improved outcomes when compared to siRNAs or plasmid shRNAs, especially in vivo [215]. Yao et al. (2012) targeted the conserved 2B region using plasmid and lentiviral vectors. Lentiviral delivery (Lenti-2B) was particularly effective in vivo, reducing viral titers, inflammation, and improving survival. The antiviral mechanism included direct RNA degradation and interference with the anti-apoptotic functions of the virus [216]. Additional work explored 5′ UTR targeting using chemically modified siRNAs (siLNAs and gapmers), with LNA-siRNA No. 20 and viral titers were significantly reduced, and cell viability was improved [217,218]. AAV-mediated delivery of shRNAs targeting 3D^pol^ achieved >1000-fold viral reduction in cardiomyocytes and improved heart function in vivo [219]. Stein et al. (2015) showed that combining AAV-shRNA targeting RdRp with sCAR-Fc further improved cardiac protection and reduced viral load in a myocarditis model [220]. Luan et al. (2012) confirmed that siRNAs targeting 2C and 3C of CV-B3 were more effective than those targeting structural regions like VP1 or the 5′ UTR, identifying siRNA-5 (targeting 2C) as especially potent [221]. Another study developed a Dicer-processed pool of siRNAs from a long dsRNA spanning the 2B-3D regions, achieved a strong cross-inhibition of CV-B3, CV-B4, and CV-A9 without IFN activation [222]. Werk et al. (2009) demonstrated that combining sCAR-Fc with siRNAs targeting 3D^pol^ synergistically reduced viral load and improved cell survival in a persistent CV-B3 model [223].

For CV-B4, Tan et al. (2010) tested siRNAs targeting conserved non-structural genes (2A, 3C, 3D). siRNA 3C showed the strongest effect, reducing replication significantly, followed by siRNA 3D. No cytotoxicity or IFN activation was observed, and antiviral effects lasted up to 48 h. Combining siRNAs yielded no additive benefit, but the study confirmed the therapeutic potential of siRNA 3C [224]. In the case of echovirus 30, Rothe et al. (2009) used computational modeling to design potent siRNAs against the 3D^pol^ gene, with several showing strong antiviral activity and one achieving an IC_50_ of ~1 nM. Additionally, targeting the host factor decay-accelerating factor (DAF) with RNAi partially inhibited infection. Combining shRNAs against both 3D^pol^ and DAF using adeno-associated virus (AAV) for delivery produced stronger and more durable inhibition [225]. In a related study, they developed a modular AAV-compatible shRNA expression system capable of targeting multiple genes simultaneously. Dual-targeting vectors against RdRp and DAF achieved enhanced suppression of viral replication, even though additive effects were not always observed. This strategy also enabled future expansion to 4–6 shRNAs per vector [226].

For EV-A71, Tan et al. (2007) demonstrated that 19-mer siRNAs and plasmid-expressed shRNAs targeting 3D^pol^ protected suckling mice from infection and paralysis without inducing IFN responses. In contrast, longer 29-mer shRNAs were potent in vitro but failed in vivo, likely due to processing inefficiency [227]. In a follow-up study, 29-mer shRNAs targeting 3D^pol^, 3C^pro^, and 2C were evaluated. The shRNA against 3D^pol^ was most effective (91% inhibition), with no cytotoxicity or IFN activation. Enhanced potency was attributed to better processing or Dicer binding [228]. Deng et al. (2012) evaluated both unmodified and chemically modified siRNAs (2′-O-Me, 2′-F) against the 5′ UTR of EV-A71. These siRNAs suppressed viral RNA, protein expression, and cytopathic effects in RD cells without triggering immune responses, suggesting their clinical promise [229]. Liu et al. (2016) designed three siRNAs against the conserved 2A^pro^ region of EV-A71, all of which showed strong antiviral effects against multiple strains in vitro, highlighting the 2A protease as a viable therapeutic target [230]. Li et al. (2013) uncovered that miR-548 family members negatively regulated IFN-λ1 expression by binding to the 3′ UTR. Inhibition of miR-548 increased IFN-stimulated gene expression and reduced EV-A71 replication, making the miR-548 inhibitor a potential therapeutic option [231]. Fang et al. identified the 3A protein of EV-A71 as a viral suppressor of RNAi (VSR). Peptides like ER-DRI neutralized this VSR function, restored RNAi, and suppressed infection in cells and mice, validating VSR-targeting as a novel antiviral strategy [232].

For EV-70, two siRNAs targeting 3D^pol^ significantly suppressed viral RNA and protein levels in infected RD cells without activating the IFN pathway. si-3D2 was more effective than si-3D1 and showed both prophylactic and therapeutic effects in vitro [233]. Jun et al. (2011) later developed AHCe-3D-3, a cross-reactive siRNA targeting the 3D^pol^ gene conserved in both EV-70 and CV-A24, which are the etiological agents of acute hemorrhagic conjunctivitis. AHCe-3D-3 was effective in primary human conjunctival cells and showed cytoprotective effects against both viruses. The accessibility to its target region likely enhanced its potency, and the siRNA retained efficacy regardless of 5′ phosphorylation status [234]. For CV-A16, Wu et al. (2008) screened 30 siRNAs targeting conserved regions of eight viral genes. Thirteen candidates could reduce reporter activity by >80%, and several significantly suppressed viral replication in Vero cells. A pooled siRNA mixture also proved effective and safe, showing dose-dependent inhibition with no cytotoxicity. The results supported the use of multi-siRNA cocktails to prevent resistance and expand antiviral coverage [235].

Collectively, these studies underscore the versatility and promise of RNAi-based strategies against a wide spectrum of EVs. The success of these approaches hinged on selecting conserved, functionally essential target sites, optimizing delivery systems (e.g., AAV, lentivirus), and in some cases, combining viral and host-targeted strategies to improve durability and suppress viral escape. Despite progress, challenges remained in mammalian antiviral RNAi research. These include inconsistent detection of vsiRNAs, limited cleavage efficiency of full-length Dicer, and the lack of a clear phenotype in Dicer-deficient cells, in terms of increased viral replication. Addressing these limitations is essential for advancing RNAi-based antiviral strategies against EVs and other RNA viruses.

### 5.3. CRISPR-Based Antivirals

The CRISPR-Cas system, originally discovered as a bacterial adaptive immune mechanism, uses RNA-guided nucleases to recognize and cleave specific DNA or RNA sequences. The precision and efficiency of CRISPR-Cas have made it a leading tool for genome editing in mammalian cells [236]. Since the pioneering use of Cas9, the development of Cas12 and Cas13 enzymes has broadened the CRISPR toolkit to include both DNA- and RNA-targeting applications [237]. This versatility renders CRISPR systems especially attractive for antiviral interventions, as viral genomes are composed of either DNA or RNA [238,239]. Beyond editing, catalytically inactive variants like dCas9 can be fused with regulatory domains to modulate gene expression through CRISPR interference (CRISPRi) or activation (CRISPRa) [237]. Compared to conventional gene-silencing methods such as siRNA or shRNA, CRISPR offers enhanced specificity, adaptability, and functional range, making it a powerful platform for developing novel antiviral strategies [240].

**Figure 7:**
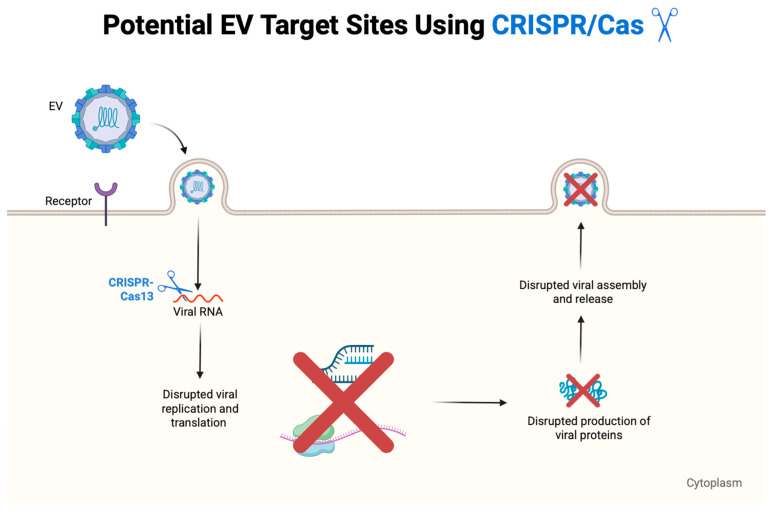
Antiviral mechanism of CRISPR-Cas13 against EVs. CRISPR-Cas13 targeted and cleaved viral RNA in the cytoplasm, disrupting replication and translation. This blocked the production of viral proteins and interfered with virion assembly and release. This approach offers a sequence-specific strategy to inhibit positive-sense RNA viruses such as EV-A71, CV-B3, and EV-D68 [237]. The figure was created using Biorender.com.

Building on these fundamental capabilities, CRISPR-Cas technology has recently been explored as a promising antiviral strategy against EVs. Given the high mutation rates and genetic plasticity of EVs, conventional antivirals often struggle with the rapid emergence of resistance. CRISPR systems, particularly Cas13, provide a unique advantage by directly targeting and degrading viral RNA with high specificity. The RNA-guided RNase activity of Cas13 enables it to cleave single-stranded viral genomes in a sequence-specific manner, without requiring a protospacer adjacent motif (PAM), making it especially suitable for targeting RNA viruses like EV-A71, CV-A16, and CV-B3 [237]. This approach is illustrated in Figure 7, which highlights the RNA-targeting activity of CRISPR-Cas13 and its interference with viral replication and release in EV-infected cells. Recent studies have demonstrated the utility of CRISPR-based screening platforms to identify host dependency factors required for EV infection and replication. These genome-wide knockout or CRISPRi screens could uncover essential cellular receptors, cofactors, and immune modulators that support viral entry or replication. Such discoveries not only illuminate virus–host interactions but also offer opportunities to design host-targeted therapeutics with reduced risk of resistance [241].

Recent studies have demonstrated the utility of CRISPR-based screening platforms to identify host dependency factors required for EV infection and replication. These genome-wide knockout or CRISPRi screens have uncovered several host proteins that facilitate viral entry and propagation. For instance, Diep et al. (2019) identified SET domain-containing 3 (SETD3), an actin histidine methyltransferase, as a key host factor for rhinovirus, EV-D68, and EV-A71. SETD3 was shown to interact with the viral 2A protease independent of its enzymatic activity—a previously unrecognized proviral role essential for EV replication [242]. Similarly, olfactomedin-like 3 (OLFML3) was found to support rhinovirus infection by suppressing the interferon response, while mannosyl (alpha-1,6-)-glycoprotein beta-1,6-N-acetyl-glucosaminyltransferase (MGAT5) and a component of the oligomeric Golgi complex subunit 1 (COG1) were shown to be critical for EV-D68 infection [243,244].

Beyond initial screens, CRISPR-Cas has proven instrumental in clarifying conflicting findings from earlier RNAi studies. The host protein, Acyl-Coenzyme A Binding Domain Containing 3 (ACBD3), previously proposed to interact with the EV 3A protein and mediate PI4KB recruitment to viral replication organelles, yielded inconsistent results when knocked down by siRNA. Some studies reported inhibition of poliovirus replication, while others observed no impact on viral replication or PI4KB localization [245,246,247,248]. To resolve this, Lyoo et al. (2019) employed CRISPR-Cas9 to generate an ACBD3 knockout cell line and conclusively demonstrated its essential role in supporting replication of EV-A71, poliovirus, and rhinovirus, as well as in PI4KB recruitment [108]. These findings highlighted the superior precision and reliability of complete gene knockout over transient knockdown approaches.

In addition to host gene perturbation, direct targeting of the viral genome using multiplexed guide RNAs has been proposed to enhance therapeutic durability. By simultaneously attacking multiple conserved regions of the viral genome, CRISPR-Cas strategies might prevent the emergence of escape mutants—a critical concern in RNA virus therapeutics [249,250]. Despite these promising developments, several challenges remained. Effective in vivo delivery of CRISPR components, particularly to tissues affected by EVs (e.g., heart, brain, or gastrointestinal tract), remains a technical bottleneck. Additionally, concerns about off-target effects, immunogenicity, and long-term safety must be rigorously addressed. Strategies such as lipid nanoparticle encapsulation, tissue-specific AAV vectors, and high-fidelity Cas variants are currently under investigation to improve delivery precision and minimize unintended consequences [251,252,253].

A recent study demonstrated the potent antiviral potential of CRISPR-Cas13d delivered via AAV against EV-A71, a major cause of HFMD for which no specific antiviral treatment currently exists. Using a custom bioinformatics pipeline called Cas13gRNAtor, researchers designed guide RNAs (gRNAs) that targeted conserved regions across the EV-A71 genome. These gRNAs were packaged with Cas13d into AAV vectors for both prophylactic and therapeutic applications. In in vitro assays, AAV-CRISPR-Cas13d constructs reduced EV-A71 viral titers by more than 99.99%. When administered to lethally infected mice, the treatment significantly inhibited viral replication, prevented clinical symptoms, and dramatically improved survival rates. Notably, the CRISPR-Cas13d system effectively cleared the virus from critical tissues such as the spinal cord and skeletal muscle, areas where siRNA-based approaches previously failed to access. The gRNA pool strategy—targeting multiple conserved sites within the viral 3D polymerase gene—was shown to enhance antiviral efficacy and maintain broad activity across various EV-A71 strains. Importantly, whole transcriptome analysis confirmed that this approach was highly specific, with no detectable off-target effects. Compared to RNAi, the AAV-CRISPR-Cas13d platform offered superior tissue penetration, durability, and viral clearance. This study highlighted the feasibility of using AAV-delivered CRISPR-Cas13 as a DAA modality for RNA viruses like EV-A71, with strong potential for further development into clinically applicable treatments for EV infections [254].

Altogether, CRISPR-Cas technology offers a transformative framework for antiviral intervention against EVs. Its dual capacity to manipulate host factors and directly cleave viral RNA positions it as a next-generation antiviral platform, capable of overcoming limitations associated with traditional RNAi or small-molecule drugs. Continued refinement of delivery systems, guided RNA design, and an understanding of viral escape mechanisms will be essential to translate this potential into clinical application.

### 5.4. Peptide-Based Antivirals

Peptides are increasingly recognized as viable therapeutic agents, with a growing number under investigation as antimicrobial and antiviral compounds in clinical trials [255]. Antiviral peptides (AVPs) are typically cationic and amphipathic, allowing them to interact with both viral and host components. These physicochemical features support the rational design of peptides that inhibit viral entry or fusion, often by mimicking sequences derived from the virus itself [256,257].

Compared to small molecules, peptides offer advantages such as high specificity, reduced off-target effects, better tolerability, and minimal toxic byproducts, as their degradation products are amino acids. Moreover, peptides are generally less susceptible to resistance since they can target multiple functional regions of the virus across different stages of the life cycle. Combining AVPs that act on different mechanisms may further reduce the likelihood of viral escape [258]. However, only one peptide-based antiviral, enfuvirtide, is currently FDA-approved for HIV, where it inhibits viral fusion with host cells [259]. Other peptide drugs, such as vancomycin, bacitracin, and neosporin, are widely used against bacterial infections [260].

A major challenge in peptide-based antiviral development is the high cost of production and formulation. Issues such as low oral bioavailability, rapid enzymatic degradation, short plasma half-life, and poor systemic delivery limit the therapeutic utility of natural peptides. For instance, enfuvirtide requires twice-daily injections, costing around USD 90/day, which impacts patient adherence. Strategies such as truncating peptides to identify minimal active motifs, recombinant peptide expression, and the use of gold nanoparticles or delivery technologies (e.g., PharmaFilm™) have been explored to overcome these barriers [258].

Peptides also hold promise in combating drug resistance. Small-molecule antivirals often induce resistant mutants, as seen with influenza and RSV. For example, RSV developed escape mutations (P488I/V, D486N, D498Y, L141W) against GS-5806, RV-521, and JNJ-53718678 [261,262,263]. In contrast, peptides such as 3ac targeting RSV’s post-fusion 6B complex retained efficacy against these mutants [264]. Likewise, EV-A71 rapidly develops resistance to small-molecule antivirals like ribavirin (via S264L, G64R/T in 3D^pol^) and capsid-binding imidazolidinones (via I113M, V123I in VP1) [265,266].

To bypass such resistance, targeting viral entry via host receptors with short peptides offers a compelling approach. SP40, derived from EV-A71 VP1, was shown to block viral attachment by interacting with the nucleolin receptor [267,268,269]. Another example is RGDS, which inhibits fibronectin receptor-mediated entry of EV-A71 [270]. SP81, a synthetic peptide derived from the VP1 capsid protein of EV-A71, demonstrated potent antiviral activity with low cytotoxicity (CC_50_: 90.32 µM). It interfered with multiple stages of infection, including viral attachment, entry, and post-entry replication, and also exhibited direct virucidal effects, achieving approximately 95% inactivation within 5 min. The peptide is predicted to interact with viral capsid proteins such as VP1 and may also inhibit IRES-mediated translation. Although in vivo stability remains a limitation, strategies such as D-amino acid substitution or nanocarrier delivery could improve its therapeutic application [271]. These peptides, though promising, still require optimization to improve stability, systemic bioavailability, and delivery before clinical translation. Overall, peptide-based antivirals represent a promising but underdeveloped class of therapeutics for enteroviruses and other RNA viruses. Continued advances in peptide design, delivery technologies, and formulation strategies are expected to enhance their clinical potential.

## 6. Challenges in Antiviral Development for the Treatment of EV Infections

Despite increasing recognition of EVs as significant human pathogens, particularly EV-D68, which has been associated with outbreaks of acute flaccid myelitis (AFM), there are still no approved antiviral therapies. While several compounds such as Rupintrivir, Enviroxime, and Pleconaril have shown efficacy against EV-D68 in vitro, they have not progressed to clinical application [272]. The lack of approved treatments is partly due to the rarity and unpredictable nature of EV-D68, causing severe infections, making it difficult to conduct well-planned randomized controlled trials. The sporadic and geographically dispersed pattern of outbreaks further complicated efforts to systematically evaluate potential therapies [59].

A key limitation in the past was the absence of reliable animal models for studying EV-D68 pathogenesis and therapeutic responses. This has been addressed in part through the development of a cotton rat model using non-adapted virus, and more notably, neonatal mouse models that displayed EV-D68-induced paralysis—an important advance for evaluating candidate treatments for AFM [195,273]. More recent work has introduced a respiratory disease model using 4-week-old AG129 mice infected with a mouse-adapted EV-D68 strain. This model reproduces key aspects of human infection, including robust viral replication in the lungs and dissemination to other tissues, and it offers a cost-effective, physiologically relevant platform for antiviral screening [272]. However, modeling the rare AFM phenotype remained a significant hurdle. Even under optimized experimental conditions, large cohorts are often required to observe sufficient cases of paralysis, which limits the efficiency of preclinical studies. Strategies such as using immunodeficient mice or adapting the virus to enhance neurovirulence have improved model sensitivity, but the lack of standardized protocols across research groups continues to hinder data comparability and broader application [274].

These challenges are not limited to EV-D68. Other clinically relevant EVs, such as EV-A71 and CV-B3, also face comparable hurdles in the development of effective therapeutics. One major obstacle is the inconsistent manifestation of disease in animal models. For example, EV-A71 infection in neonatal mice often fails to replicate hallmark features of severe human disease, such as brainstem encephalitis, neurogenic pulmonary edema, or limb paralysis [275]. Similarly, CV-B3-induced myocarditis in mice is highly strain- and age-dependent, with common outcomes like pancreatitis that are not typically seen in human patients, thereby reducing the translational value of these models. Reproducing the full clinical spectrum—including acute neurological or cardiac complications—remained a significant challenge. Although the use of neonatal animals, genetically modified mice, or virus-adapted strains has improved model sensitivity and enabled the study of specific disease mechanisms, these approaches still fall short in recapitulating the complex pathophysiology observed in humans. Moreover, many models only represent the acute phase of infection, lacking the capacity to study long-term complications or chronic sequelae [276]. In addition, the limited availability of harmonized and standardized protocols across research laboratories impeded consistent evaluation of antiviral efficacy. Variability in animal models, infection routes, and disease scoring criteria complicated cross-study comparisons and contributed to the lack of consensus regarding the predictive value of preclinical data. Collectively, these issues underscore the urgent need for better experimental systems that more faithfully mimic human disease and can support rigorous testing of antiviral candidates across a broader range of EVs.

In parallel, structure-based drug design (SBDD) approaches are gaining traction. One study focused on a compound that was structurally similar to fluoxetine, but lacked serotonin reuptake inhibitor activity, which showed broad-spectrum inhibition of EV-D68, EV-A71, and CV-A24v by targeting the conserved 2C protein. Another study employed virtual screening to identify quinoline analogs, with compound **19** exhibiting potent activity across multiple EV-D68 strains. It functioned primarily by binding to the VP1 capsid protein’s hydrophobic pocket, effectively inhibiting viral protein expression and replication in vitro. This compound also displayed good bioavailability in rats and metabolic stability in human liver microsomes [63,92]. Target-directed drug design (TDD), which relies on structural insights from crystallography and homology modeling, has also been applied to identify antiviral candidates and optimize existing leads. By comparing wild-type and resistant viral protein structures, TDD facilitated the rational development of drugs that retained efficacy against emerging variants [277].

In conclusion, while important advances have been made in modeling EV infections and identifying candidate antivirals, numerous challenges persist. These include the infrequent occurrence of severe disease outcomes across various EV serotypes, the need for validated and standardized animal models that could reflect diverse disease manifestations, and the difficulty of translating promising in vitro results into clinical success. Continued efforts in refining experimental systems, applying computational drug design, and leveraging innovative molecular technologies would be essential to accelerate the discovery and development of effective antiviral therapies against EVs.

## 7. Conclusions

EVs represent a significant public health burden due to their wide range of clinical manifestations, from mild febrile illness to severe neurological and cardiac complications. Despite substantial research efforts, there are still no approved antiviral therapies targeting EV infections. This therapeutic gap is largely driven by challenges such as high viral mutation rates, limited broad-spectrum efficacy, and poor translation from in vitro studies to effective in vivo outcomes.

Numerous small-molecule antivirals have been explored, targeting viral capsid proteins, proteases, and replication complexes. While several compounds have shown potent in vitro activity, many failed to progress due to resistance, toxicity, or narrow strain specificity. Recent advances in structure-based drug design and high-throughput screening have yielded promising leads, but these require further optimization and clinical validation. Targeting host factors presents an alternative strategy with the potential to overcome viral resistance and broaden therapeutic coverage. However, this approach carries the risk of off-target effects and toxicity due to the essential role of host proteins in normal cellular functions. A detailed understanding of virus–host interactions remained critical for identifying viable targets with minimal adverse consequences. Immunotherapeutic interventions, including monoclonal antibodies, IVIG, and modulation of innate immune pathways, have also shown encouraging preclinical efficacy. These approaches highlighted the value of leveraging host immunity as a means to control EV infections. At the same time, emerging technologies such as RNAi and CRISPR-Cas systems offered new modalities that directly target viral RNA with high specificity, demonstrating robust antiviral activity both in vitro and in vivo.

Nonetheless, several overarching challenges continued to impede progress. Animal models often exhibit inconsistent disease phenotypes and limited relevance to human pathology, complicating the evaluation of candidate antivirals. The lack of standardized experimental methodologies further hampered cross-study comparisons and reproducibility. Moreover, the sporadic and geographically dispersed nature of severe EV outbreaks posed logistical difficulties for conducting designed clinical trials. Moving forward, a combination of strategies would be essential. Rational drug design, host-targeted approaches, and gene-editing technologies should be integrated with improved disease models and harmonized evaluation criteria. Combination therapies, designed to enhance efficacy and limit resistance, also hold promise for future development.

Ultimately, bridging the gap between bench and bedside would require interdisciplinary collaboration, sustained investment in antiviral research, and the continued refinement of translational tools. As some EVs remain better studied than others, extending these advances to lesser-known serotypes would be key to achieving comprehensive and durable antiviral solutions across the EV spectrum.

## Figures and Tables

**Table 1:** Direct-acting antivirals against EVs.

Target Site	Representative Compounds	EV Species	Mechanism of Action	Efficacy (In Vitro)	Notable Insights	References
VP1 hydrophobic pocket	Pleconaril, Vapendavir, PR66, BPROZ-101, NLD-22, VP1-6g	EV-A71, CV-B3, Echoviruses, EV-D68	Capsid stabilization; blocked uncoating	EC_50_/IC_50_: nM–μM	High resistance risk (Taiwan EV-A71 strains), good breadth for EV-A71, poor for EV-D68	[10,31,42,43,44,46,47,48,49]
	Compound 36, 10 g	CV-B3, CV-B4	Effective against pleconaril-resistant strains; orally bioavailable	IC_50_: 0.01–1 μM	Stable; protective in mice	[55,56]
	Compound 19 (quinoline analog)	EV-D68	Inhibited uncoating	EC_50_: 0.4–4 μM	Broad strain coverage; good pharmacokinetics profile	[34,63]
VP1-VP3 interprotomer binding pocket	4EDMAB, Compound 1, 17, 7a	CV-B3, CV-B1/B4/B5/B6, CV-A9	Stabilized virion; blocked uncoating	EC_50_: 0.7–9.4 μM	Effective across EV-B and some EV-C/D; resistance mapping ongoing	[9,64,65]
Five-fold axis of the capsid	MADL385, CB-30	EV-A71	Blocked receptor attachment	EC_50_: 0.2–353 nM	Resistance mutations (e.g., VP1-S184T); dendrimeric design	[66,67]
	Rosmarinic acid, Suramin, E151	EV-A71, CV-A16, CV-A6	Entry inhibition; blocked receptor interaction	EC_50_: 31–114 μM	Suramin effective in animal models, but limited by non-specificity	[32,37,68,69,70]
	NF110, NM16 (Suramin analogs)	EV-A71	Blocked binding to sulfated receptors (e.g., PSGL-1)	EC_50_/IC_50_: low to sub-μM	Site-specific resistance (e.g., K244R)	[69]

**Table 2:** Non-structural protein inhibitors against EVs.

Target Protein	Representative Compounds	EV Species	Mechanism of Action	Notable Insights	References
2A protease	Z-LVLQTM-FMK, CW33, Telaprevir, Chlorogenic acid	EV-A71, EV-D68	Inhibited viral polyprotein processing; blocked host translation/immune responses	Limited potency; structure-based studies emerging	[71,72,73,74,75,76,77,78,79,80,81,82]
2B protein	DIDS	EV-A71	Blocked chloride ion channel; viroporin inhibition	Unclear specificity; selective inhibitors needed	[83,84,85]
2C protein	Fluoxetine, compound 12b, dibucaine, 6aw, JX040, R523062	EV-A71, EV-D68, CV-B3, Poliovirus	Inhibited ATPase/oligomerization; disrupted replication/encapsidation	Broad-spectrum potential; structural studies advancing	[86,87,88,89,90,91,92,93,94,95,96,97,98,99,100,101,102,103]
3A protein	Enviroxime, AN-12-H5, itraconazole, TTP-8307	EV-A71, EV-D68, CV-B3	Disrupted replication organelle formation	No confirmed direct binders; promising polypharmacology	[105,106,107,108,109,110,111,112,113,114,115,116,117,161]
3C protease	Rupintrivir, AG7404, compound 18p, NK-1.9k	EV-A71, EV-D68, CV-B3, Echoviruses	Inhibited viral proteolytic processing; immune evasion	Potent in vitro activity; resistance and delivery issues remained	[118,119,120,121,122,123,124,125,126,127,128,129,130,131,132,133,134,135,136,137,138,139,140,141,142,143,144,145,146,162]
3D polymerase	Gemcitabine, LY2334737, FNC, GPC-N114, BPR-3P0128, DTriP-22	EV-A71, CV-B3, EV-D68, Poliovirus	Chain termination; RdRp inhibition; blocked RNA elongation	Broad-spectrum potential; nucleoside analogs in clinical use	[147,148,149,150,151,152,154,156,157,158,159,160]

**Table 3:** Host-targeting antivirals against EVs.

Host Target	Representative Compounds/Strategies	Mechanism of Action	Antiviral Effects	References
OSBP (Oxysterol-binding protein)	OSW-1, Itraconazole, TTP-8307, T-00127-HEV2	Disrupted cholesterol transport; impaired viral replication complex formation	Potent EV-A71 inhibition; nanomolar EC_50_ values; reduced OSBP levels by ~90%	[166,167,168,169,170]
PI4KB (Phosphatidylinositol 4-kinase IIIβ)	MDL-860, Compound 10, PIK93, T-00127-HEV1	Inhibited PI4KB function and lipid kinase activity; blocked replication organelle formation	Nanomolar activity against CV-B3 and EV-A71; MDL-860 analog showed 50% protection in neonatal mice	[171,172,173]
DHODH (Dihydroorotate dehydrogenase)	RYL-634, FA-613	Inhibited pyrimidine biosynthesis; disrupted viral RNA synthesis	RYL-634: EC_50_ ~4 nM (EV-A71); FA-613: Broad activity against RSV, EVs, CoVs	[174,175,176,177]
Innate Immune Modulators (e.g., TLR7, IFN pathways)	R837 (TLR7 agonist), IFN-α, GS-9620	Activated antiviral IFN responses; reduced viral replication/inflammation	Improved survival in EV-A71 mouse models; synergistic with rupintrivir	[178,179,180]
Restriction Factors (e.g., SAMHD1, APOBEC3G)	Endogenous upregulation or stabilization	SAMHD1 blocked VP1–VP2 interaction; A3G disrupted 5′ UTR translation initiation.	Inhibited EV-A71, CV-A16, and EV-D68	[181,182,183,184]
Attachment Factors (e.g., HSPGs)	HTA-22, heparin, lactoferrin	Blocked viral attachment	Inhibited EV-A71, CV-A16, and PV1	[185,186,187,188]
hNMT1 (N-myristoyltransferase 1)	siRNA knockdown, Compound 4O	Prevented VP4 myristoylation; impaired capsid precursor processing.	Inhibited EV-A71 replication	[22]
AP2M1 (Adaptor protein complex 2 subunit mu)	ACA (N-(p-amylcinnamoyl)anthranilic acid)	Disrupted 2C localization; inhibited YxxØ motif	Inhibited EV-A71, influenza, Zika, and MERS-CoV.	[189]
mTOR signaling and autophagy	Torin2, LY-55	Inhibited mTOR-mediated autophagy	Inhibited EV-A71 replication with low IC_50_ values; synergistic with 3-MA.	[190,191]
TREM-1–NF-κB–MAPK axis	LP17 peptide	Suppressed virus-induced inflammatory cytokine signaling.	Reduced IL-6, IL-8, TNF-α release in EV-D68-infected cells.	[192]
Cyclophilin A (CypA)	Cyclosporine A, HL051001P2, CypA-11	Blocked VP1–CypA interaction, impaired uncoating.	Exhibited submicromolar activity against EV-A71; synergistic with 3C^pro^ inhibitors.	[193,194]
